# Supplementary material for: Rapid metabolic shifts occur during the transition between hunger and satiety in Drosophila melanogaster
Source: Nat Commun. 2019 Sep 6;10:4052. doi: 10.1038/s41467-019-11933-z (PMC6731244; doi:10.1038/s41467-019-11933-z)
Supplement: Supplementary file 1 — Supplementary Information [file 41467_2019_11933_MOESM1_ESM.pdf]

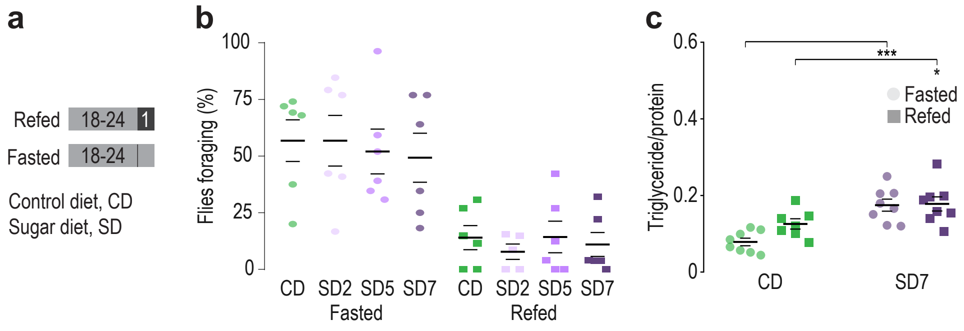
a
Refed
18-24
1
Fasted
18-24
Control diet, CD
Sugar diet, SD
b
Flies foraging (%)
100
75
50
25
0
CD
SD2
SD5
SD7
CD
SD2
SD5
SD7
Fasted
Refed
c
Triglyceride/protein
0.6
0.4
0.2
0
***
*
Fasted
Refed
CD
SD7

**Supplementary Fig. 1 The foraging behavior of fasted and refed flies on a control or high sugar diet.**

**a)** Schematic of feeding manipulations used in the manuscript. Flies were food deprived for 18-24 hr then given access to vials containing 1 % agar only (fasted, lighter gray) or 1 % agar with 400 mM D-Glucose agar (refed, black) for 1 hr. After this time, flies were immediately used for metabolomics, transcriptomics, or behavioral experiments as described in the Methods.

**b)** The foraging behavior of flies fed a control diet (CD, green), or high sugar diet (SD) for 2 days (SD2, lavender), 5 days (SD5, lighter purple), and 7 days (SD7, darker purple) and then fasted (lighter shades) or refed (darker shades) as shown in a). Data are mean (thicker line) +/- standard error of the mean (SEM, thinner lines) for this figure. Two-way ANOVA with Dunnett's test. n = 6 (biologically independent samples) vials of 21-27 age-matched male flies.

**c)** The triglyceride measurements normalized to protein of flies on a CD (green) and SD7 (darker purple) then fasted (lighter shades) or refed (darker shades). Data are mean +/- SEM for this panel. Two-way ANOVA with Sidak's test: * $p < 0.05$, *** $p < 5 \times 10^{-4}$. SD7 flies and CD fasted n = 8 and CD refed n = 7 biologically independent animals. Source data are provided as a Source Data file.

Wilinski et al.
Supplementary Fig. 2

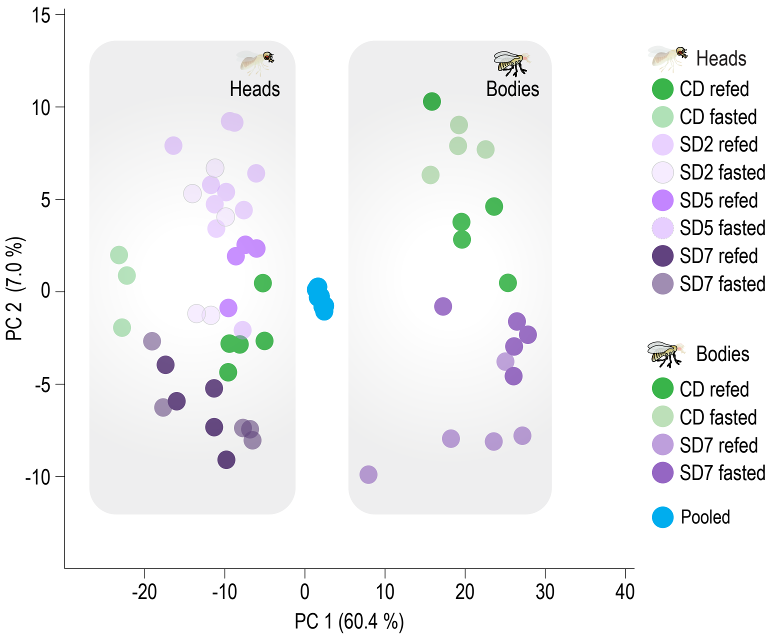

**Supplementary Fig. 2 Principal component analysis of metabolomics data.** Projection onto the two most explanatory principal components for the fly head and body samples used in the metabolomics study. Control diet (CD, green shades); high sugar diet (SD2, SD5, or SD7 days, purple shades); pooled (blue) refers to samples that combined all the replicates shown. Percent variance is listed in parentheses.

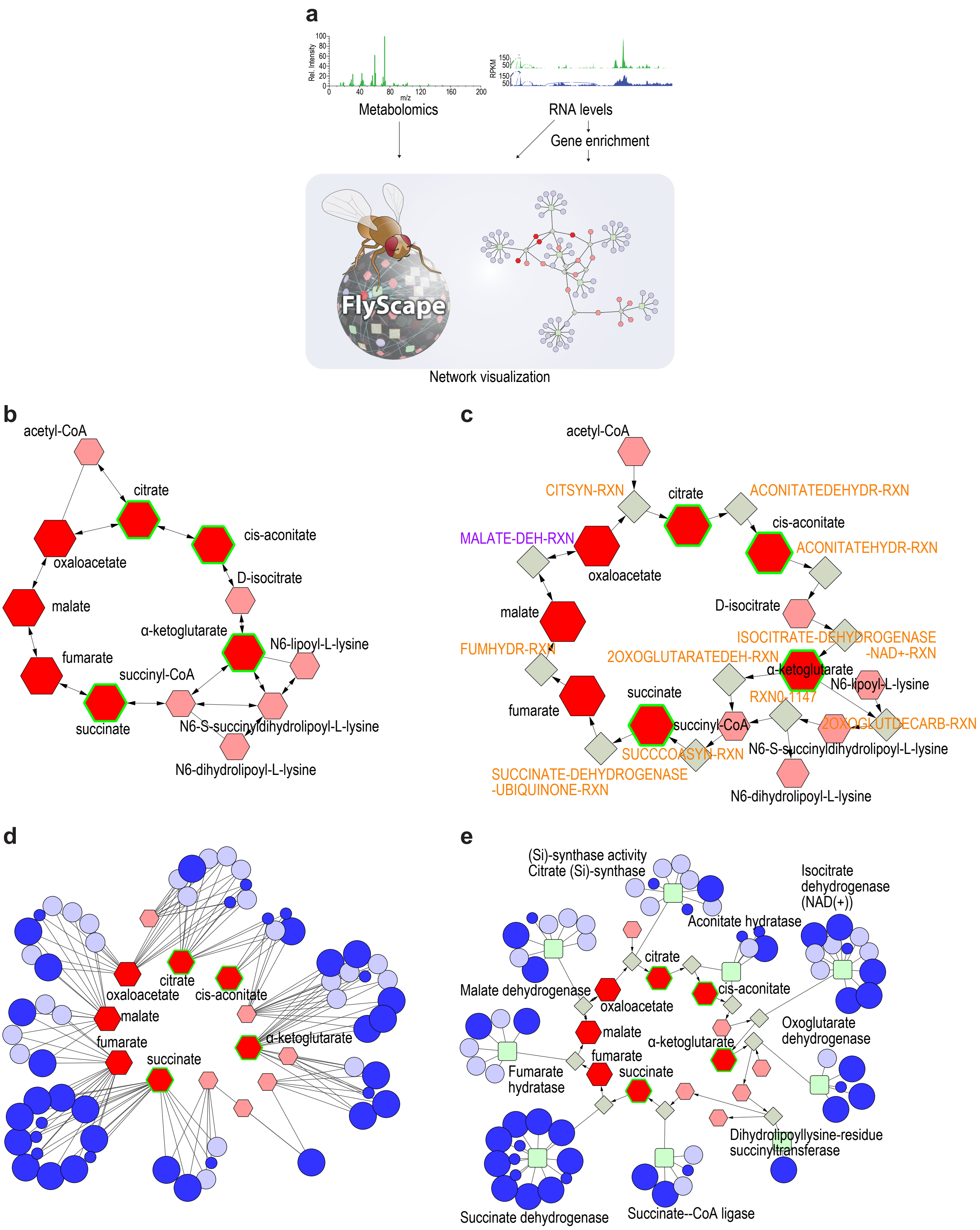
a
Rel. Intensity
m/z
RPKM
Metabolomics
RNA levels
Gene enrichment
FlyScape
Network visualization
b
acetyl-CoA
citrate
cis-aconitate
oxaloacetate
D-isocitrate
malate
α-ketoglutarate
N6-lipoyl-L-lysine
fumarate
succinyl-CoA
succinate
N6-S-succinyldihydrolipoyl-L-lysine
N6-dihydrolipoyl-L-lysine
c
acetyl-CoA
CITSYN-RXN
citrate
ACONITATEDEHYDR-RXN
cis-aconitate
MALATE-DEH-RXN
ACONITATEHYDR-RXN
oxaloacetate
malate
D-isocitrate
ISOCITRATE-DEHYDROGENASE
-NAD+-RXN
FUMHYDR-RXN
2OXOGLUTARATEDEH-RXN
α-ketoglutarate
N6-lipoyl-L-lysine
succinate
RXN0-1147
fumarate
succinyl-CoA
2OXOGLUTDECARB-RXN
SUCCCOASYN-RXN
N6-S-succinyldihydrolipoyl-L-lysine
SUCCINATE-DEHYDROGENASE
-UBIQUINONE-RXN
N6-dihydrolipoyl-L-lysine
d
citrate
oxaloacetate
cis-aconitate
malate
fumarate
α-ketoglutarate
succinate
e
(Si)-synthase activity
Citrate (Si)-synthase
Isocitrate
dehydrogenase
(NAD(+))
Aconitate hydratase
citrate
Malate dehydrogenase
cis-aconitate
oxaloacetate
malate
Oxoglutarate
dehydrogenase
α-ketoglutarate
fumarate
Fumarate
hydratase
succinate
Dihydrolipoyllysine-residue
succinyltransferase
Succinate dehydrogenase
Succinate--CoA ligase

**Supplementary Fig. 3 Flyscape, a tool to map and visualize *Drosophila melanogaster* metabolic networks.**

**a)** Diagram of workflow that Flyscape uses to analyze metabolomic and transcriptomic data.

**b-e)** Examples of different Flyscape compound networks visualization where compounds are represented as nodes and reactions are represented as edges **(b)**, compound-reaction **(c),** compound-gene **(d)**, and compound-reaction-enzyme-gene networks **(e).** The size of the compound nodes (red hexagons) reflects changes in metabolite abundance (up or down), salmon-colored hexagons represent compounds that were not measured, the size of the gene nodes (blue circles) represents the magnitude of changes in RNA abundance (up or down), light blue circles are genes that were not measured, green squares represent the enzyme type, and the gray diamonds are reactions. Source data are provided in the Source Data file.

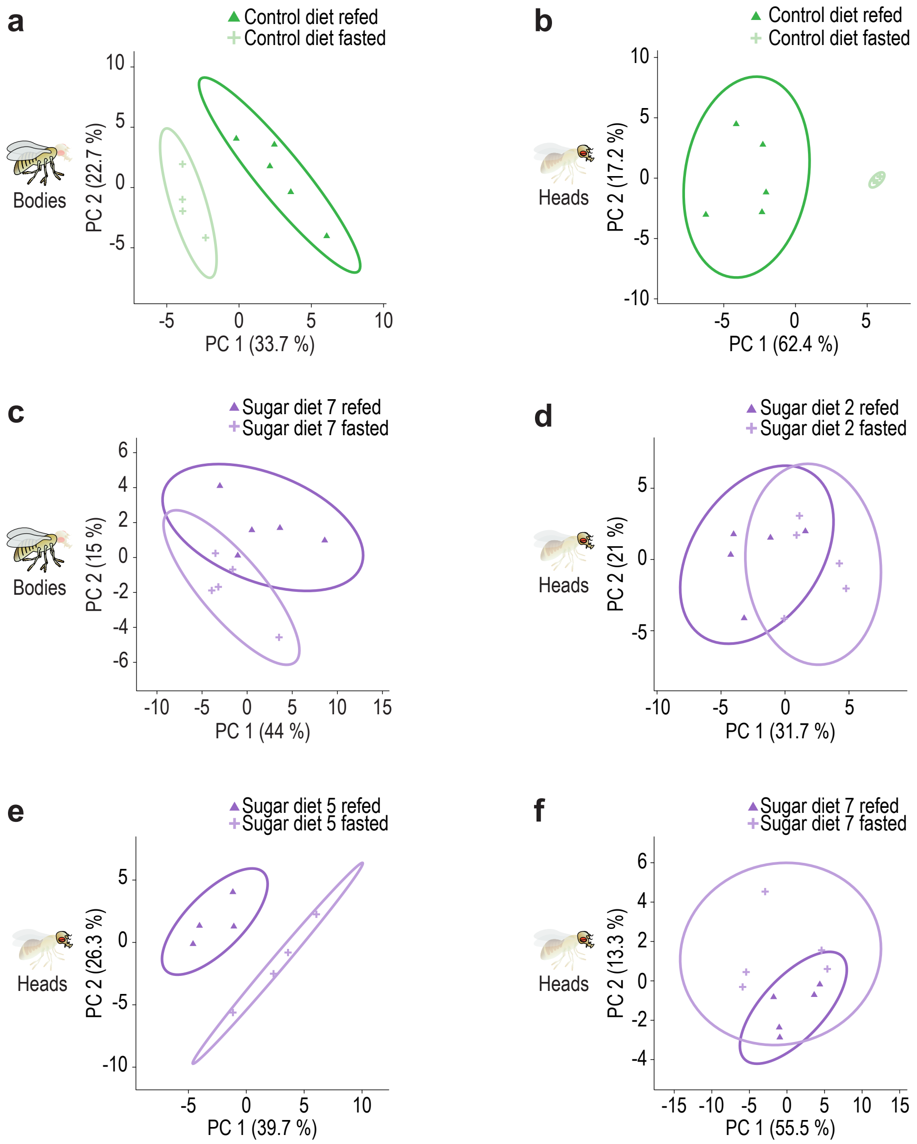
a
Control diet refed
Control diet fasted
Bodies
PC 2 (22.7 %)
PC 1 (33.7 %)
b
Control diet refed
Control diet fasted
Heads
PC 2 (17.2 %)
PC 1 (62.4 %)
c
Sugar diet 7 refed
Sugar diet 7 fasted
Bodies
PC 2 (15 %)
PC 1 (44 %)
d
Sugar diet 2 refed
Sugar diet 2 fasted
Heads
PC 2 (21 %)
PC 1 (31.7 %)
e
Sugar diet 5 refed
Sugar diet 5 fasted
Heads
PC 2 (26.3 %)
PC 1 (39.7 %)
f
Sugar diet 7 refed
Sugar diet 7 fasted
Heads
PC 2 (13.3 %)
PC 1 (55.5 %)

**Supplementary Fig. 4 Principal component analysis of metabolomics samples by tissue, feeding state, and diet.**

**a-f)** Projections onto the two strongest principal components for metabolomics samples **a)** refed and fasted bodies fed a control diet (CD), **b)** refed and fasted heads fed a CD, **c)** refed vs fasted bodies fed a high sugar diet (SD7), and refed and fasted heads fed a SD for **d)** 2 days (SD2), **e)** 5 days (SD5), or **f)** 7 days (SD7). Percent variance is listed in parentheses and the circles represent the 95 % confidence interval. Source data are provided in the Source Data file.

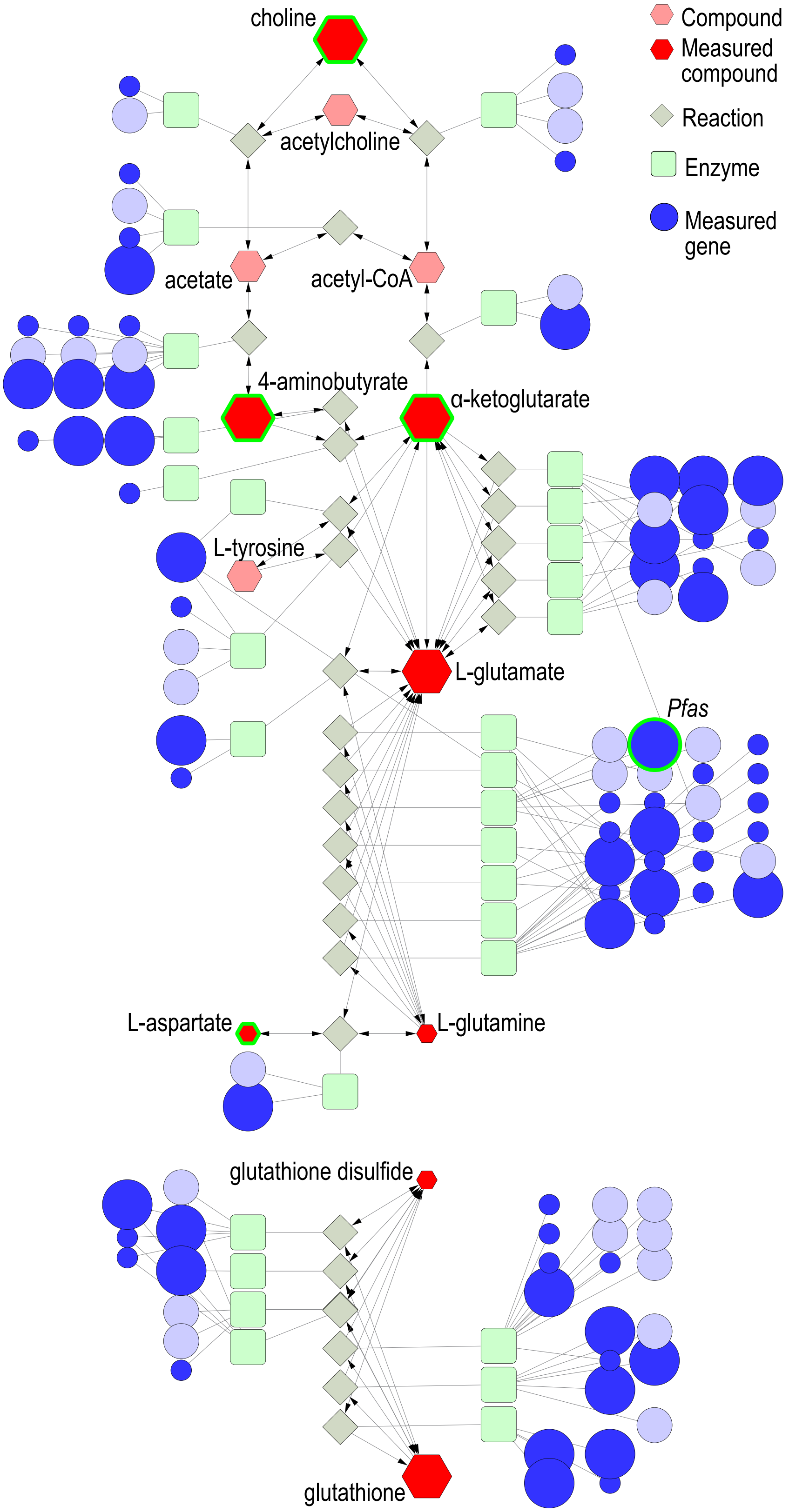
choline
acetylcholine
acetate
acetyl-CoA
4-aminobutyrate
α-ketoglutarate
L-tyrosine
L-glutamate
Pfas
L-aspartate
L-glutamine
glutathione disulfide
glutathione
Compound
Measured compound
Reaction
Enzyme
Measured gene

**Supplementary Fig. 5 The metabolite levels of neurotransmitter intermediates change between the fasted and refed states in fruit fly heads.**

Flyscape network showing changes in neurochemicals from the heads of refed and fasted flies fed a control diet. FDR < 0.1 by Welch's t test for metabolites and FDR < 0.05 by Wald test for RNA sequencing (green outlines). The size of the compound nodes (red hexagons) reflects changes in metabolite abundance (up or down) between fasted and refed fly heads, (salmon-colored hexagons represent compounds that were not measured), the size of the gene nodes (blue circles) represents the magnitude of changes in RNA abundance (up or down) between fasted and refed brains, light blue circles are genes that were not measured, green squares represent the enzyme type, and the gray diamonds are reactions. Source data are provided in the Source Data file.

**a**

Brains

| Sample Name | Condition | % Aligned | M Aligned | Length | M Seqs |
|---|---|---|---|---|---|
| 91819_AGTGAG_S18_R1 | Control diet refed | 79.6 | 11.2 | 38 | 14.1 |
| 91819_AGTGAG_S18_R2 | Control diet refed | | | 38 | 14.1 |
| 91820_GCACTA_S19_R1 | Control diet sated | 78.0 | 9.7 | 38 | 12.5 |
| 91820_GCACTA_S19_R2 | Control diet sated | | | 38 | 12.5 |
| 91821_ACCTCA_S20_R1 | Control diet sated | 75.2 | 9.6 | 38 | 12.8 |
| 91821_ACCTCA_S20_R2 | Control diet sated | | | 38 | 12.8 |
| 91822_GTGCTT_S21_R1 | Control diet fasted | 74.0 | 9.2 | 38 | 12.4 |
| 91822_GTGCTT_S21_R2 | Control diet fasted | | | 38 | 12.4 |
| 91823_AAGCCT_S22_R1 | Control diet fasted | 72.6 | 9.1 | 38 | 12.5 |
| 91823_AAGCCT_S22_R2 | Control diet fasted | | | 38 | 12.5 |
| 91826_AGCATG_S25_R1 | Control diet refed | 79.3 | 11 | 38 | 13.9 |
| 91826_AGCATG_S25_R2 | Control diet refed | | | 38 | 13.9 |
| 91827_GAGTCA_S26_R1 | Control diet refed | 79.6 | 9.9 | 38 | 12.5 |
| 91827_GAGTCA_S26_R2 | Control diet refed | | | 38 | 12.5 |
| 91828_CGTAGA_S27_R1 | Control diet sated | 79.4 | 11.1 | 38 | 14 |
| 91828_CGTAGA_S27_R2 | Control diet sated | | | 38 | 14 |
| 91830_CACAGT_S29_R1 | Control diet fasted | 80.3 | 11 | 38 | 13.7 |
| 91830_CACAGT_S29_R2 | Control diet fasted | | | 38 | 13.7 |

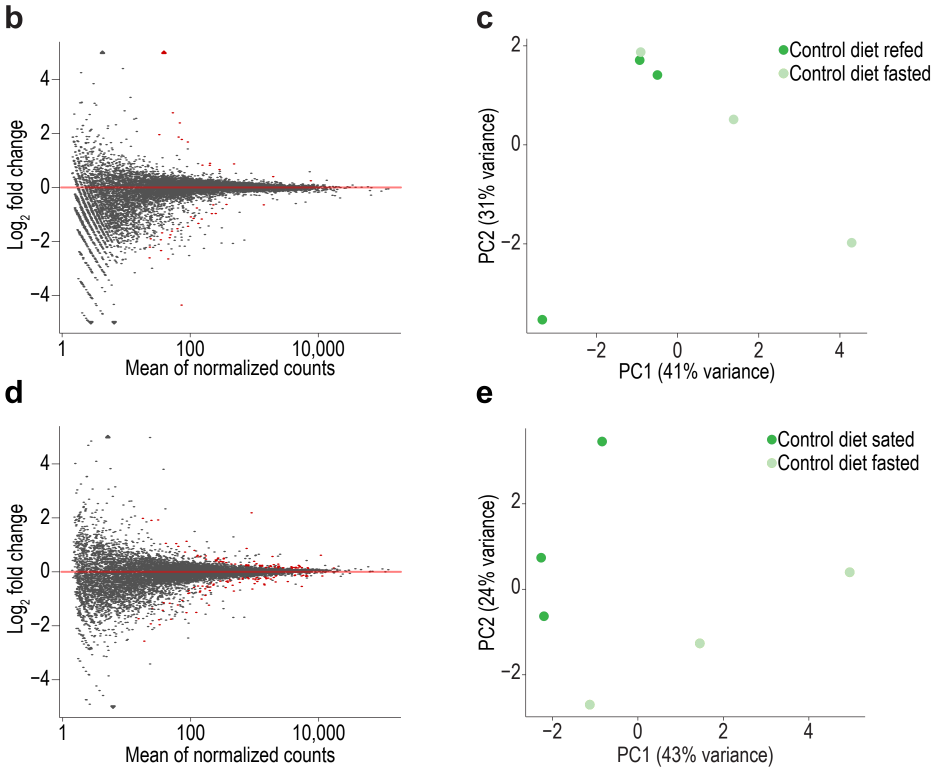

**Supplementary Fig. 6 Analysis of RNA-sequencing from the brains of fasted, refed, and sated flies.**

**a)** Summary of sequencing reads analysis by sample.

**b** and **d)** MA plots of the comparison between the brains of: **b)** fasted and refed flies, and **d)** fasted and sated flies. Red circles in MA plot FDR < 0.05 by Wald test.

**c and e)** Projection onto the two most explanatory principal components for the fasted, refed, and sated RNA sequencing samples. Source data are provided as a Source Data file.

Wilinski et al.
Supplementary Fig. 7

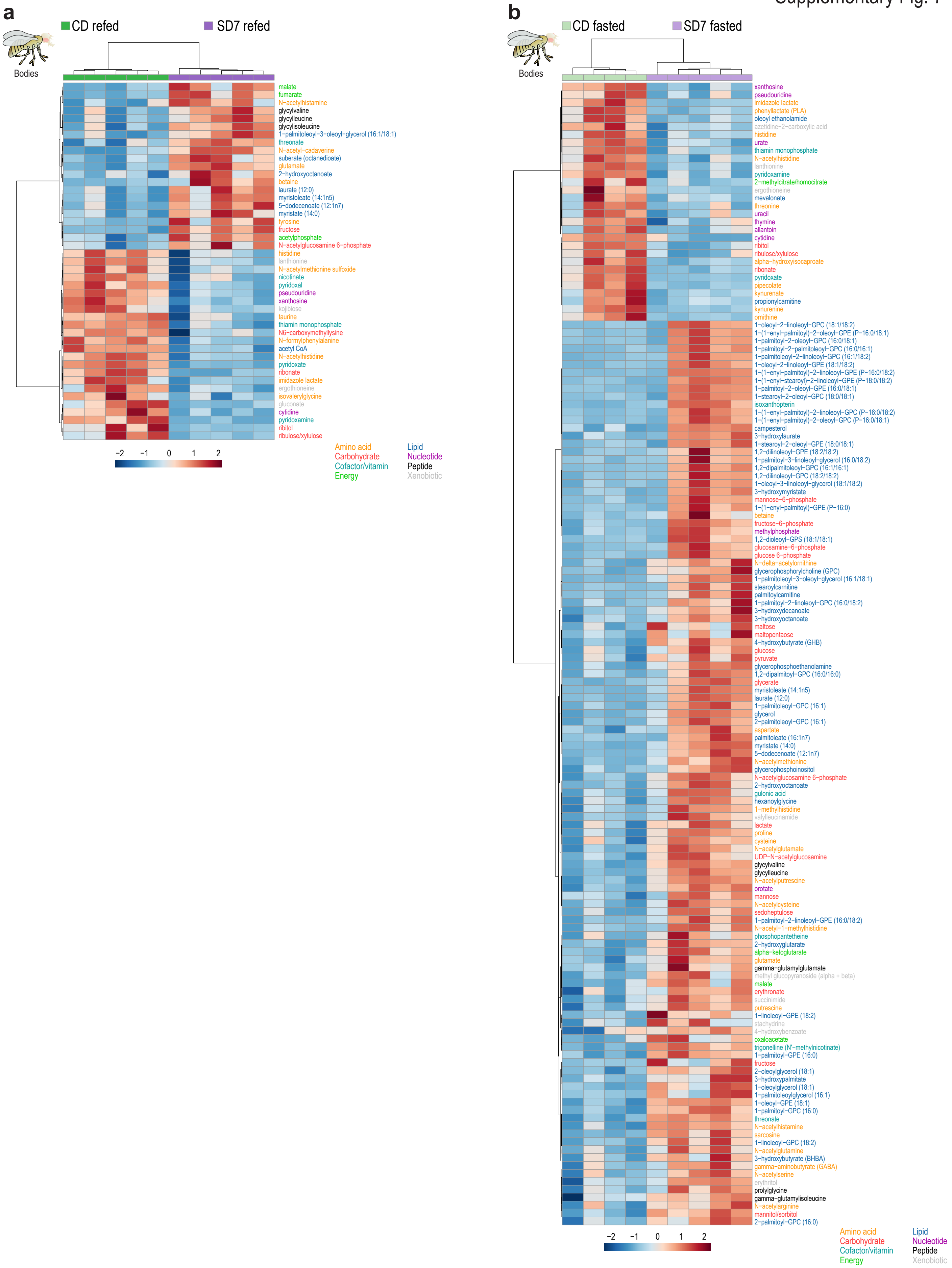

**Supplementary Fig. 7 Comparison of metabolites that change in the bodies of refed and fasted flies fed a control or high sugar diet.**

Heatmap representing the **a)** 45 metabolic compounds that change in the bodies of refed flies between a control diet (CD) and a high sugar diet for 7 days (SD7) and **b)** the 145 metabolic compounds that change in the bodies of fasted flies between a CD and SD7. Welsh test, FDR < 0.1. Normalized compound levels were clustered by compound (rows) and data replicate (columns). Metabolite names are colored according to class (bottom). The heatmap indicates positive (red shades) and negative (blue shades) normalized compound levels. Source data are provided as a Source Data file.

Wilinski et al.
Supplementary Fig. 8

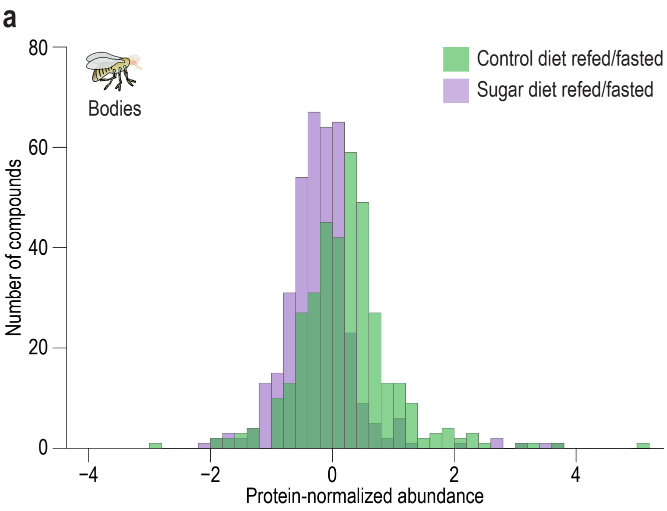


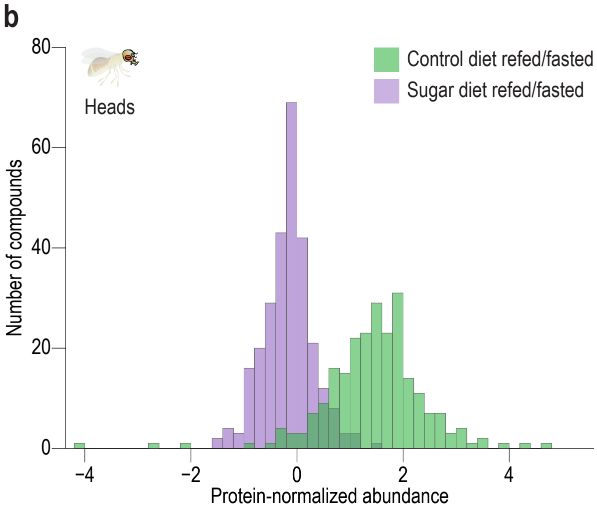

**Supplementary Fig. 8 Distribution of fold changes in metabolites between refed and fasted fly bodies and heads**.

**a, b)** Histogram of compound $\log_2$ fold changes in protein-normalized abundance between the refed and fasted conditions on control (CD, green) or high sugar diet for 7 days (SD7, purple) for **a)** bodies and **b)** heads. Source data are provided in the Source Data file.

**a**

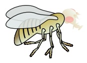

Bodies

| Sample Name | Condition | % Aligned | M Aligned | Length | M Seqs |
|---|---|---|---|---|---|
| ERR1717103_1 | Sugar diet | 95 | 17.8 | 75 | 18.7 |
| ERR1717103_2 | Sugar diet | | | 74 | 18.7 |
| ERR1717104_1 | Sugar diet | 95.5 | 19.2 | 75 | 20.1 |
| ERR1717104_2 | Sugar diet | | | 74 | 20.1 |
| ERR1717105_1 | Sugar diet | 95.7 | 19.6 | 75 | 20.5 |
| ERR1717105_2 | Sugar diet | | | 74 | 20.5 |
| ERR1717110_1 | Control diet | 95.7 | 21 | 75 | 21.9 |
| ERR1717110_2 | Control diet | | | 74 | 21.9 |
| ERR1717111_1 | Control diet | 96.1 | 19.3 | 75 | 20.1 |
| ERR1717111_2 | Control diet | | | 74 | 20.1 |
| ERR1717112_1 | Control diet | 96.3 | 20.1 | 75 | 20.8 |
| ERR1717112_2 | Control diet | | | 74 | 20.8 |

**b**

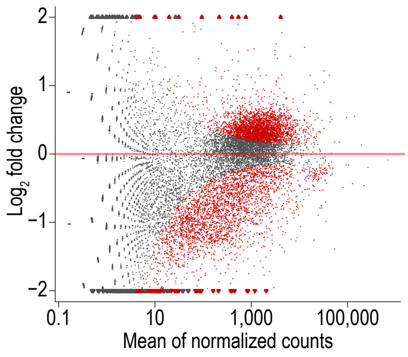


**c**

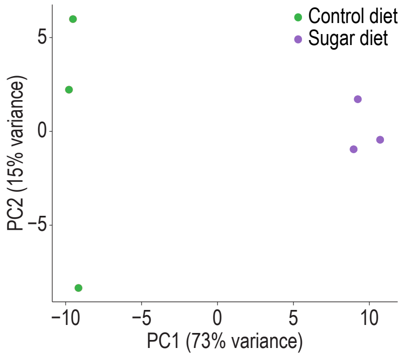

**Supplementary Fig. 9 Summary of RNA-sequencing of fly bodies.**

Reanalysis of RNA sequencing of flies fed a control or sugar from a previously published study[1].

**a)** Summary of sequencing reads; **b)** MA plot; **c)** Projection onto the two most explanatory principal components of the samples from a). Red circles in MA plot FDR $< 0.05$ by Wald test. Source data are provided as a Source Data file.

Wilinski et al.
Supplementary Fig. 10

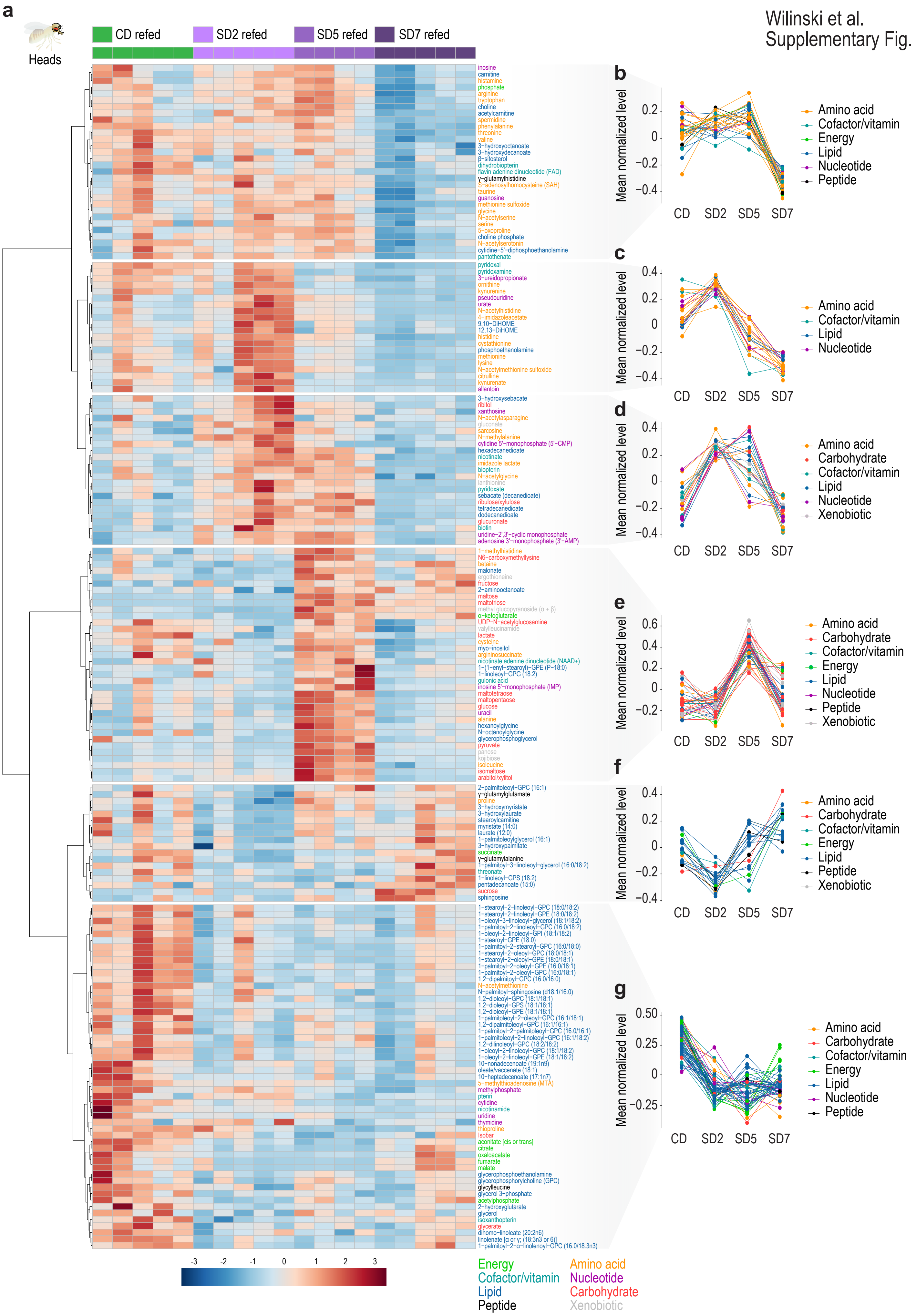

**Supplementary Fig. 10 Comparison of metabolites that change in the heads of sated flies fed a control or high sugar diet for different days.**

**a)** Heatmap representing the 180 metabolic compounds changed in the heads of refed flies on a control diet (CD, green) and high sugar diet for 2, 5, or 7 days (SD2, SD5, SD7, shades of purple); ANOVA, FDR $< 0.1$. Normalized compound levels were clustered by compound (rows). Metabolite names are colored according to class (bottom).

**b-g)** The mean normalized levels of each compound across each condition (CD, SD2, SD5, and SD7) found in each of the 6 clusters in a). The heatmap indicates positive (red shades) and negative (blue shades) normalized compound levels. Names are colored based on compound class (right). Source data are provided as a Source Data file.

Wilinski et al.
Supplementary Fig. 11

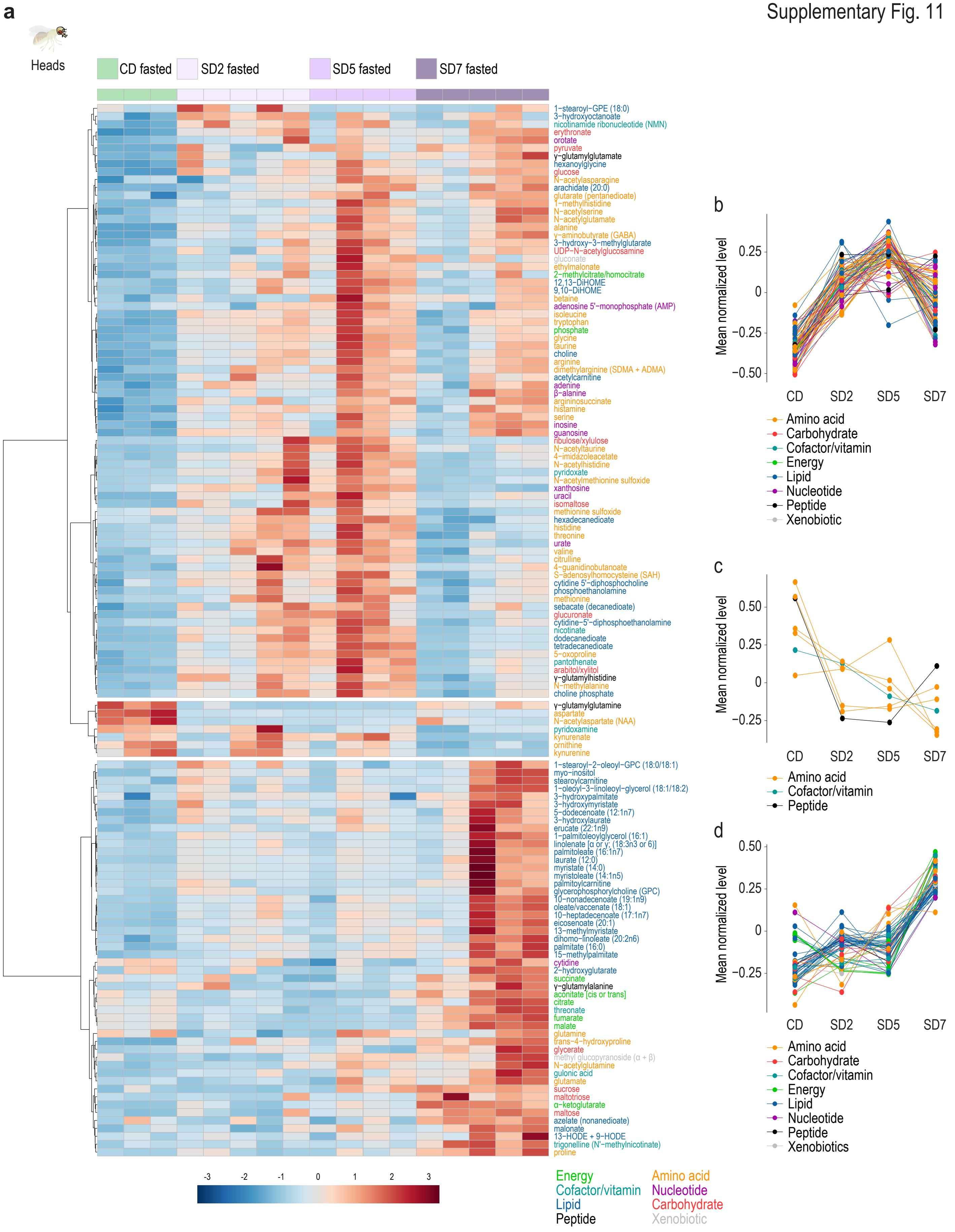

**Supplementary Fig. 11 Comparison of metabolites that change in the heads of fasted flies fed a control or high sugar diet for different days.**

**a)** Heatmap representing the 132 metabolic compounds changed in the heads of fasted flies between a control diet (CD, green) and high sugar diet SD for 2, 5, or 7 days (SD2, SD5, SD7, shades of purple). ANOVA, FDR < 0.1. Normalized compound levels were clustered by compound (rows). Metabolite names are colored according to class (bottom).

**b-d)** The mean normalized levels of each compound across conditions (CD, SD2, SD5, and SD7) found in each of the 3 clusters in (a). The heatmap indicates positive (red shades) and negative (blue shades) normalized compound levels. Names are colored based on compound class (right). Source data are provided as a Source Data file.

**Supplementary Note 1**

R code used to analyze raw FLIC data.

# FLIC Analysis for Figure 1

R scripts reference site: https://github.com/PletcherLab/FLIC_R_Code/tree/master/FLIC

For more information see: https://journals.plos.org/plosone/article?id=10.1371/journal.pone.0101107

### Getting started

Change the working directory to the folder which contains the data and R scripts.

```
knitr::opts_chunk$set(echo = TRUE)
knitr::opts_knit$set(root.dir = "Y:/Wilinski, Daniel/Metabolomics/FLICData/Figure 1")
```

Before getting started a few packages may need to be installed. RStudio will output the error message detailing any missing packages.

### Load source scripts:

```
source("SingleWellChamber.R")
```

```
## Loading required package: ggplot2
```

```
## Warning: package 'ggplot2' was built under R version 3.5.3
```

```
source("TastingDataSum.Well.R")
source("AvgTasteDur.Well.R")
source("CountTastingEvents.Well.R")
source("FeedingAndTasting.Summary.Monitors.R")
source("FeedingAndTasting.Summary.OneWell.R")
source("FeedingAndTasting.Summary.R")
source("GetLatency.R")
source("GetTastingEvents.Well.R")
source("ParametersClass.R")
source("SingleWellChamber.R")
source("TastingDataSum.Well.R")
source("DFM.R")
source("CommonChamber.R")
```

### Set-up parameters for single well experiments

```
p<-ParametersClass.SingleWell()
```

The following parameters need to be set and were determined empirically to represent feeding and tasting.

```
p<-SetParameter(p,Feeding.Threshold.Value=40)
p<-SetParameter(p,Feeding.Interval.Minimum=40)
p<-SetParameter(p,Tasting.Threshold.Interval=c(10,40))
```

### Output summary data

Create the summary file of feeding and tasting data for the DFMs tested. The DFMs are listed within “c()” - these numbers should be exactly as they are output by the MCU. For example, DFM_1 would be listed below as “1”. Specify the start and end time (in minutes) for data analysis within “range=c(0,60)”. The start time cannot be modified from “0”.

```
FeedingAndTasting.Summary.Monitors(p,c(200, 3, 4, 9, 11, 14, 21, 1900, 4000, 5000, 2700),range=c(0,60))
```

The output file is called “FeedingSummary_DFM” followed by the numbers of the first and last DFMs listed in the previous line. Using the above as an example, the output file would be called “FeedingSummary_DFM200_2700”

# FLIC Analysis for Figure 5

R scripts reference site: https://github.com/PletcherLab/FLIC_R_Code/tree/master/FLIC

For more information see: https://journals.plos.org/plosone/article?id=10.1371/journal.pone.0101107

### Getting started

Change the working directory to the folder which contains the data and R scripts.

```
knitr::opts_chunk$set(echo = TRUE)
knitr::opts_knit$set(root.dir = "Y:/Wilinski, Daniel/Metabolomics/FLICData/Figure 5")
```

Before getting started a few packages may need to be installed. RStudio will output the error message detailing any missing packages.

### Load source scripts:

```
source("SingleWellChamber.R")
```

```
## Loading required package: ggplot2
```

```
## Warning: package 'ggplot2' was built under R version 3.5.3
```

```
source("TastingDataSum.Well.R")
source("AvgTasteDur.Well.R")
source("CountTastingEvents.Well.R")
source("FeedingAndTasting.Summary.Monitors.R")
source("FeedingAndTasting.Summary.OneWell.R")
source("FeedingAndTasting.Summary.R")
source("GetLatency.R")
source("GetTastingEvents.Well.R")
source("ParametersClass.R")
source("SingleWellChamber.R")
source("TastingDataSum.Well.R")
source("DFM.R")
source("CommonChamber.R")
```

### Set-up parameters for single well experiments

```
p<-ParametersClass.SingleWell()
```

The following parameters need to be set and were determined empirically to represent feeding and tasting.

```
p<-SetParameter(p,Feeding.Threshold.Value=40)
p<-SetParameter(p,Feeding.Interval.Minimum=40)
p<-SetParameter(p,Tasting.Threshold.Interval=c(10,40))
```

### Output summary data

Create the summary file of feeding and tasting data for the DFMs tested. The DFMs are listed within "c()" - these numbers should be exactly as they are output by the MCU. For example, DFM_1 would be listed below as "1". Specify the start and end time (in minutes) for data analysis within "range=c(0,60)". The start time cannot be modified from "0".

```
FeedingAndTasting.Summary.Monitors(p,c(1, 2, 3, 4, 5, 6, 7, 8, 9, 11, 12, 13, 14, 15, 16, 17, 18, 19, 2[illegible]
```

The output file is called “FeedingSummary_DFM” followed by the numbers of the first and last DFMs listed in the previous line. Using the above as an example, the output file would be called “FeedingSummary_DFM1_400”

**Supplementary Note 2**

R code used for statistical analysis of metabolomics data.

# Metaboanalyst analysis (pruned pairwise comparisions)

## Import data (Metabolon)

We want to check the raw data to make sure we are on using comopunds with only imputed values. So we need to look at the raw data for missing values. We also need the normalized data from the previous analysis

```
library(tidyverse)
```

```
## Warning: package 'tidyverse' was built under R version 3.5.3
```

```
## -- Attaching packages ----------------------------------------------------------------------------
```

```
## v ggplot2 3.1.0       v purrr   0.3.2
## v tibble  2.1.1       v dplyr   0.8.0.1
## v tidyr   0.8.3       v stringr 1.4.0
## v readr   1.3.1       v forcats 0.4.0
```

```
## Warning: package 'ggplot2' was built under R version 3.5.3
```

```
## Warning: package 'tibble' was built under R version 3.5.3
```

```
## Warning: package 'tidyr' was built under R version 3.5.3
```

```
## Warning: package 'readr' was built under R version 3.5.3
```

```
## Warning: package 'purrr' was built under R version 3.5.3
```

```
## Warning: package 'dplyr' was built under R version 3.5.3
```

```
## -- Conflicts --------------------------------------------------------------------------------------
## x dplyr::filter() masks stats::filter()
## x dplyr::lag()    masks stats::lag()
```

```
library(MetaboAnalystR)
```

```
## Loading required package: lattice
```

```
## Loading required package: pls
```

```
##
## Attaching package: 'pls'
```

```
## The following object is masked from 'package:stats':
##
##     loadings
```

```
data <- read.csv("metabolonOrginalData.csv", header = TRUE, row.names = 5)
# normData <- read.csv('data_normalized_RangeNorm.csv')
dir.create("analysis")
dir.create("analysis/dataprocessed")
dir.create("analysis/normdata")
dir.create("analysis/ttest")
dir.create("analysis/foldchange")
dir.create("analysis/pca")
dir.create("analysis/heatmap")
```

### Make lists of data columns

Each pairwise comparsion using the following data sets.

```
ND_bodies <- c("a7D_ND_sated_bodies_1",
               "a7D_ND_sated_bodies_2",
               "a7D_ND_sated_bodies_3",
               "a7D_ND_sated_bodies_4",
               "a7D_ND_sated_bodies_5",
               "a7D_ND_starved_bodies_6",
               "a7D_ND_starved_bodies_7",
               "a7D_ND_starved_bodies_8",
               "a7D_ND_starved_bodies_9",
              # "a7D_ND_starved_bodies_10"
              5,4)
ND_heads <- c("a7D_ND_sated_heads_6",
              "a7D_ND_sated_heads_7",
              "a7D_ND_sated_heads_8",
              "a7D_ND_sated_heads_9",
              "a7D_ND_sated_heads_10",
              "a7D_ND_starved_heads_1",
              "a7D_ND_starved_heads_2",
              "a7D_ND_starved_heads_3",
              #"a7D_ND_starved_heads_4",
              5,3)
SD7_bodies <- c("a7D_HSD_sated_bodies_21",
                "a7D_HSD_sated_bodies_22",
                "a7D_HSD_sated_bodies_23",
                "a7D_HSD_sated_bodies_24",
                "a7D_HSD_sated_bodies_25",
                "a7D_HSD_starved_bodies_26",
                "a7D_HSD_starved_bodies_27",
                "a7D_HSD_starved_bodies_28",
                "a7D_HSD_starved_bodies_29",
                "a7D_HSD_starved_bodies_30",
                5,5)
SD2_heads <- c("a2D_HSD_sated_heads_41",
               "a2D_HSD_sated_heads_42",
               "a2D_HSD_sated_heads_43",
               "a2D_HSD_sated_heads_44",
               "a2D_HSD_sated_heads_45",
               "a2D_HSD_starved_heads_46",
               "a2D_HSD_starved_heads_47",
               "a2D_HSD_starved_heads_48",
               "a2D_HSD_starved_heads_49",
               "a2D_HSD_starved_heads_50",
               5,5)
SD5_heads <- c(#"a5D_HSD_sated_heads_31",
               "a5D_HSD_sated_heads_32",
               "a5D_HSD_sated_heads_33",
               "a5D_HSD_sated_heads_34",
               "a5D_HSD_sated_heads_35",
               "a5D_HSD_starved_heads_36",
               "a5D_HSD_starved_heads_37",
               "a5D_HSD_starved_heads_38",
```

```
                "a5D_HSD_starved_heads_39",
                #"a5D_HSD_starved_heads_40",
                4,4)
SD7_heads <- c("a7D_HSD_sated_heads_11",
                "a7D_HSD_sated_heads_12",
                "a7D_HSD_sated_heads_13",
                "a7D_HSD_sated_heads_14",
                "a7D_HSD_sated_heads_15",
                "a7D_HSD_starved_heads_16",
                "a7D_HSD_starved_heads_17",
                "a7D_HSD_starved_heads_18",
                "a7D_HSD_starved_heads_19",
                "a7D_HSD_starved_heads_20",
                5,5)
NDvSD_sated_bodies <- c("a7D_ND_sated_bodies_1",
                "a7D_ND_sated_bodies_2",
                "a7D_ND_sated_bodies_3",
                "a7D_ND_sated_bodies_4",
                "a7D_ND_sated_bodies_5",
                "a7D_HSD_sated_bodies_21",
                "a7D_HSD_sated_bodies_22",
                "a7D_HSD_sated_bodies_23",
                "a7D_HSD_sated_bodies_24",
                "a7D_HSD_sated_bodies_25",
                5,5)
NDvSD_staved_bodies <- c("a7D_ND_starved_bodies_6",
                "a7D_ND_starved_bodies_7",
                "a7D_ND_starved_bodies_8",
                "a7D_ND_starved_bodies_9",
                "a7D_HSD_starved_bodies_26",
                "a7D_HSD_starved_bodies_27",
                "a7D_HSD_starved_bodies_28",
                "a7D_HSD_starved_bodies_29",
                "a7D_HSD_starved_bodies_30",
                4,5)
```

## Pariwise analysis

### Subset the data into chuncks that for pariwise comparison

Remove the comounds (rows) that did were not measure in the orginal data.

```
listofvectors <- list(ND_bodies, ND_heads, SD7_bodies, SD2_heads, SD5_heads,
    SD7_heads, NDvSD_sated_bodies, NDvSD_staved_bodies)
# getwd()
```

This creates a list of vectors (c(something) concatenates the vectors into one so you get each individual element as one)

```
for(dataname in listofvectors) {
#data_subset <- data %>%
#  select (dataname)

## preprocessing step
    data.subset <- data %>%
```

```r
    # select(dataname)  %>%
    select(dataname[1:(length(dataname)-2)])  %>% ## take only a subset of the data
    rownames_to_column("COMP_ID") #%>% ## make row names a column
    #slice(2:n()) ## remove top line
row.vector1 <- (as.numeric())
row.vector2 <- (as.numeric())
for (row in 1:nrow(data.subset)) {
  temp <- data.subset[row,]
  # split temp into two vectors based on number of replicates
  temp1 <- temp[2:((as.numeric(dataname[(length(dataname)-1)]))+1)]
  temp2 <- temp[((as.numeric(dataname[(length(dataname)-1)]))+2):((as.numeric(dataname[length(dataname)[illegible]

  #print (rowSums(is.na(temp)))
  row.vector1 <- c(row.vector1, rowSums(is.na(temp1)))
  row.vector2 <- c(row.vector2, rowSums(is.na(temp2)))
}
data.subset.na <- cbind(row.vector1, row.vector2, data.subset)

## define the number of NAs allowed in each data set
rm(cutoff.nas)

dim.data1 <- (as.numeric(dataname[(length(dataname)-1)]))
dim.data2 <- (as.numeric(dataname[(length(dataname))]))
## first set
if(dim.data1 == 3) {
  cutoff.nas1 <- 2
}
if(dim.data1 == 4) {
  cutoff.nas1 <- 3
}
if(dim.data1 == 5) {
  cutoff.nas1 <- 3
}
## second set
if(dim.data2 == 3) {
  cutoff.nas2 <- 2
}
if(dim.data2 == 4) {
  cutoff.nas2 <- 3
}
if(dim.data2 == 5) {
  cutoff.nas2 <- 3
}

# remove rows that exceed the number of NAs
data.subset.na.cutoff <- dplyr::filter(data.subset.na, row.vector1 < cutoff.nas1)
data.subset.na.cutoff <- dplyr::filter(data.subset.na.cutoff, row.vector2 < cutoff.nas2)

# remove row.vector column
data.subset.na.cutoff.n <- dplyr::select(data.subset.na.cutoff, -row.vector1, -row.vector2)


## end preprocessing
```

```
write.csv(data.subset.na.cutoff.n,file="tmp_data_subset.csv",row.names = FALSE)

rm (mSet)  ## clear data
mSet<-InitDataObjects("conc", "stat", FALSE)
#par(family="sans")
mSet<-Read.TextData(mSet, "tmp_data_subset.csv", "colu", "disc");
mSet<-SanityCheckData(mSet)
mSet$dataSet$check.msg

mSet<-RemoveMissingPercent(mSet, percent=0.5)   ##  remove compounds that have too many missing values
mSet<-ImputeVar(mSet, method="min")
mSet$dataSet$check.msg

mSet<-Normalization(mSet, "NULL", "NULL", "RangeNorm", ratio=FALSE, ratioNum=20)

mSet<-PlotNormSummary(mSet, paste("plots/norm_2_",sep=''), "png", 300, width=NA)
mSet<-PlotSampleNormSummary(mSet, paste("plots/snorm_2_",sep=''), "png", 300, width=NA)

mSet<-SaveTransformedData(mSet)

file.rename ("data_processed.csv",paste("analysis/dataprocessed/data_processed",dataname[1],dataname[len
file.rename ("data_normalized.csv",paste("analysis/normdata/data_normalized_RangeNorm_",dataname[1],data

## T-tests
  mSet<-Ttests.Anal(mSet, F, 1, FALSE, TRUE) ## I do this so that I get all the values not just signifi
  file.rename("t_test.csv",paste("analysis/ttest/t_test_",dataname[1],dataname[length(dataname)-2],".csv

## Fold change
  mSet<-FC.Anal.unpaired(mSet, fc.thresh= 1.0, cmp.type = 0)
  mSet<-SaveTransformedData(mSet)
  file.rename("fold_change.csv",paste("analysis/foldchange/fold_change_",dataname[1],dataname[length(dat
  #file.remove("data_original.csv")

## PCA analysis
  mSet<-PCA.Anal(mSet)
  mSet<-PlotPCA2DScore(mSet, paste ("analysis/pca/pca_score2d_",dataname[1],dataname[length(dataname)-2]
#  mSet<-PlotPCA2DScore(mSet, paste ("analysis/pca/pca_score2d_",dataname[1],dataname[length(dataname)]
  file.rename("pca_loadings.csv",paste("analysis/pca/pca_loadings_",dataname[1],dataname[length(datanam
  file.rename("pca_score.csv", paste("analysis/pca/pca_score_",dataname[1],dataname[length(dataname)-2]

## remove temp files
file.remove("tmp_data_subset_remove_percent.csv")
file.remove("data_original.csv")
file.remove("tmp_data_subset.csv")


##############
########### heatmaps2
##############################

## count number of sig compounds
fdr.p <- p.adjust(mSet$analSet$tt$p.value, "fdr")
fdr.p <- as.data.frame(fdr.p)
```

```
numb.fdr.p<-NROW(dplyr::filter(fdr.p,fdr.p < 0.1))
#numb.fdr.p <- 75 ## or make static number
print ("Number of signifcant compounds FDR 0.1 :")
print (numb.fdr.p)

if(numb.fdr.p > 0) {
  var.nms <- names(sort(mSet$analSet$tt$p.value))[1:numb.fdr.p]  ## reports the x signficant compounds b
  var.inx <- match(var.nms, colnames(mSet$dataSet$norm))
  my.data <- mSet$dataSet$norm
  hc.dat <- as.matrix(my.data[, var.inx])

  colors <- rev(colorRampPalette(RColorBrewer::brewer.pal(10, "RdBu"))(256))

  clusters<-pheatmap::pheatmap(t(hc.dat),
                               fontsize = 5,clustering_distance_rows = "euclidean",
                               clustering_distance_cols = "euclidean", clustering_method = "ward.D",
                               border_color = "grey", cluster_rows = T, cluster_cols = T, scale = "row"
                               cellheight = 4.402, cellwidth = 11.709, annotation_colors = TRUE, color =
                               filename = paste("analysis/heatmap/heatmapSigONLY_",dataname[1],dataname
                               main =  paste(dataname[1],dataname[length(dataname)-2],sep='\n'))
###################################

  colortable <- read.table("H:/DusLab/data/Metabolimics/Data/Data3/colorCompoundNames/colorClassKey.txt
NameIDClasstable <- read.table("H:/DusLab/data/Metabolimics/Data/Data3/colorCompoundNames/compoundsNameI

namesforheatmap <- clusters$tree_row$labels[clusters$tree_row$order]  ## puts IDs in order
namesforheatmap <-  as.data.frame(namesforheatmap)  ## convert to data frame
names (namesforheatmap) <- "COMP_ID"  ## add a header name

namesforheatmap <- dplyr::inner_join(namesforheatmap,NameIDClasstable, by="COMP_ID")  ## only the comp_I
namesforheatmap <- dplyr::inner_join(namesforheatmap,colortable, by = "SUPER_PATHWAY")  ## add color inf

x <- namesforheatmap$BIOCHEMICAL ## take only BIOCHEMICAL (biochemical names)
y <- c(1:numb.fdr.p)  ## dummy data

a <- namesforheatmap$color

plot_data <- data.frame(x, y)

#plot_data$y <- as.numeric(as.character(plot_data$y))
#plot_data <- t(plot_data)
plot_data <-plot_data[dim(plot_data)[1]:1,] ## flip order
a <-rev(a) ## flip order
p <- ggplot(plot_data, aes(x = y, y = x)) +
  #geom_bar(stat = "identity") +
  geom_point() +
  scale_y_discrete(limits=plot_data$x) +  ## this prevents re-sorting
  theme(axis.text.y = element_text(angle = 0, hjust = 1, color = a, size = 4)) +
  ggtitle(paste(dataname[1],dataname[length(dataname)-2],sep='\n')) +
  xlab("toy data") +
  ylab("Names")

ggsave(paste("analysis/heatmap/heatmapNamesColored_",dataname[1],dataname[length(dataname)-2],".pdf",sep
```

```
}
}
```

```
## [1] "R objects intialized ..."

## [1] "A total of 369 significant features were found."

## [1] "Number of signifcant compounds FDR 0.1 :"
## [1] 61

## [1] "R objects intialized ..."

## [1] "A total of 242 significant features were found."

## [1] "Number of signifcant compounds FDR 0.1 :"
## [1] 159

## [1] "R objects intialized ..."

## [1] "A total of 373 significant features were found."

## [1] "Number of signifcant compounds FDR 0.1 :"
## [1] 14

## [1] "R objects intialized ..."

## [1] "A total of 271 significant features were found."

## [1] "Number of signifcant compounds FDR 0.1 :"
## [1] 10

## [1] "R objects intialized ..."

## [1] "A total of 280 significant features were found."

## [1] "Number of signifcant compounds FDR 0.1 :"
## [1] 43

## [1] "R objects intialized ..."

## [1] "A total of 276 significant features were found."

## [1] "Number of signifcant compounds FDR 0.1 :"
## [1] 0
## [1] "R objects intialized ..."

## [1] "A total of 363 significant features were found."

## [1] "Number of signifcant compounds FDR 0.1 :"
## [1] 45

## [1] "R objects intialized ..."

## [1] "A total of 374 significant features were found."

## [1] "Number of signifcant compounds FDR 0.1 :"
## [1] 144
```

# Metaboanalyst analysis (pruned Anova Sated and Starved comparisions)

## Import data (Metabolon)

We want to check the raw data to make sure we are on using comopunds with only imputed values. So we need to look at the raw data for missing values. We also need the normalized data from the previous analysis

```
library(tidyverse)
```

```
## Warning: package 'tidyverse' was built under R version 3.5.3

## -- Attaching packages ---------------------------------------------------------------------------------

## v ggplot2 3.1.0       v purrr   0.3.2
## v tibble  2.1.1       v dplyr   0.8.0.1
## v tidyr   0.8.3       v stringr 1.4.0
## v readr   1.3.1       v forcats 0.4.0

## Warning: package 'ggplot2' was built under R version 3.5.3

## Warning: package 'tibble' was built under R version 3.5.3

## Warning: package 'tidyr' was built under R version 3.5.3

## Warning: package 'readr' was built under R version 3.5.3

## Warning: package 'purrr' was built under R version 3.5.3

## Warning: package 'dplyr' was built under R version 3.5.3

## -- Conflicts ------------------------------------------------------------------------------------------
## x dplyr::filter() masks stats::filter()
## x dplyr::lag()    masks stats::lag()
```

```
library(MetaboAnalystR)
```

```
## Loading required package: lattice

## Loading required package: pls

##
## Attaching package: 'pls'

## The following object is masked from 'package:stats':
##
##     loadings
```

```
data <- read.csv("metabolonOrginalData.csv", header = TRUE, row.names = 5)
# normData <- read.csv('data_normalized_RangeNorm.csv')
dir.create("analysisANOVA")
```

```
## Warning in dir.create("analysisANOVA"): 'analysisANOVA' already exists
```

```
dir.create("analysisANOVA/dataprocessed")
```

```
## Warning in dir.create("analysisANOVA/dataprocessed"):
## 'analysisANOVA\dataprocessed' already exists
```

```
dir.create("analysisANOVA/normdata")
```

```
## Warning in dir.create("analysisANOVA/normdata"): 'analysisANOVA\normdata'
## already exists
```

```
dir.create("analysisANOVA/anova")
```

```
## Warning in dir.create("analysisANOVA/anova"): 'analysisANOVA\anova' already
## exists
```

```
dir.create("analysisANOVA/foldchange")
```

```
## Warning in dir.create("analysisANOVA/foldchange"):
## 'analysisANOVA\foldchange' already exists
```

```
dir.create("analysisANOVA/pca")
```

```
## Warning in dir.create("analysisANOVA/pca"): 'analysisANOVA\pca' already
## exists
```

```
dir.create("analysisANOVA/heatmap")
```

```
## Warning in dir.create("analysisANOVA/heatmap"): 'analysisANOVA\heatmap'
## already exists
```

### Make lists of data columns

Each comparsion using the following data sets.

```
sated_heads <- c(
  "a7D_ND_sated_heads_6",
              "a7D_ND_sated_heads_7",
              "a7D_ND_sated_heads_8",
              "a7D_ND_sated_heads_9",
              "a7D_ND_sated_heads_10",
  "a2D_HSD_sated_heads_41",
               "a2D_HSD_sated_heads_42",
               "a2D_HSD_sated_heads_43",
               "a2D_HSD_sated_heads_44",
               "a2D_HSD_sated_heads_45",
  "a5D_HSD_sated_heads_32",
               "a5D_HSD_sated_heads_33",
               "a5D_HSD_sated_heads_34",
               "a5D_HSD_sated_heads_35",
  "a7D_HSD_sated_heads_11",
               "a7D_HSD_sated_heads_12",
               "a7D_HSD_sated_heads_13",
               "a7D_HSD_sated_heads_14",
               "a7D_HSD_sated_heads_15"
)

starved_heads <- c(
  "a7D_ND_starved_heads_1",
              "a7D_ND_starved_heads_2",
              "a7D_ND_starved_heads_3",
```

```
  "a2D_HSD_starved_heads_46",
                "a2D_HSD_starved_heads_47",
                "a2D_HSD_starved_heads_48",
                "a2D_HSD_starved_heads_49",
                "a2D_HSD_starved_heads_50",
  "a5D_HSD_starved_heads_36",
                "a5D_HSD_starved_heads_37",
                "a5D_HSD_starved_heads_38",
                "a5D_HSD_starved_heads_39",
                #"a5D_HSD_starved_heads_40",
  "a7D_HSD_starved_heads_16",
                "a7D_HSD_starved_heads_17",
                "a7D_HSD_starved_heads_18",
                "a7D_HSD_starved_heads_19",
                "a7D_HSD_starved_heads_20"
)
```

## Analysis

**Subset the data into chuncks for comparison**

```
listofvectors <- list(sated_heads, starved_heads)
```

This creates a list of vectors (c(something) concatenates the vectors into one so you get each individual element as one)

```
for(dataname in listofvectors) {
data_subset <- data %>%
  select (dataname)
write.csv(data_subset,file="tmp_data_subset.csv")

rm (mSet)  ## clear data
mSet<-InitDataObjects("conc", "stat", FALSE)
#par(family="sans")
mSet<-Read.TextData(mSet, "tmp_data_subset.csv", "colu", "disc");
mSet<-SanityCheckData(mSet)
mSet$dataSet$check.msg

mSet<-RemoveMissingPercent(mSet, percent=0.5)   ##  remove compounds that have too many missing values
mSet<-ImputeVar(mSet, method="min")
mSet$dataSet$check.msg

mSet<-Normalization(mSet, "NULL", "NULL", "RangeNorm", ratio=FALSE, ratioNum=20)

mSet<-PlotNormSummary(mSet, paste("plots/norm_2_",sep=''), "png", 300, width=NA)
mSet<-PlotSampleNormSummary(mSet, paste("plots/snorm_2_",sep=''), "png", 300, width=NA)

mSet<-SaveTransformedData(mSet)

file.rename ("data_processed.csv",paste("analysisANOVA/dataprocessed/data_processed",dataname[1],datanam[illegible]
file.rename ("data_normalized.csv",paste("analysisANOVA/normdata/data_normalized_RangeNorm_",dataname[1[illegible]

## T-tests
  #mSet<-Ttests.Anal(mSet, F, 1, FALSE, TRUE) ## I do this so that I get all the values not just signif[illegible]
```

```
  #file.rename("t_test.csv",paste("analysisANOVA/ttest/t_test_",dataname[1],dataname[length(dataname)],

## ANOVA
  mSet<-ANOVA.Anal(mSet, F, 1.0, "fisher")
  file.rename("anova_posthoc.csv",paste("analysisANOVA/anova/anova_",dataname[1],dataname[length(datanar


## Fold change
  mSet<-FC.Anal.unpaired(mSet, fc.thresh= 1.0, cmp.type = 0)
  mSet<-SaveTransformedData(mSet)
  file.rename("fold_change.csv",paste("analysisANOVA/foldchange/fold_change_",dataname[1],dataname[lengt
  file.remove("data_original.csv")

## PCA analysis
  mSet<-PCA.Anal(mSet)
  mSet<-PlotPCA2DScore(mSet, paste ("analysisANOVA/pca/pca_score2d_",dataname[1],dataname[length(datanar
 # mSet<-PlotPCA2DScore(mSet, paste ("analysisANOVA/pca/pca_score2d_",dataname[1],dataname[length(datan
  file.rename("pca_loadings.csv",paste("analysisANOVA/pca/pca_loadings_",dataname[1],dataname[length(dat
  file.rename("pca_score.csv", paste("analysisANOVA/pca/pca_score_",dataname[1],dataname[length(dataname

## remove temp files
file.remove("tmp_data_subset_remove_percent.csv")
file.remove("data_original.csv")
file.remove("tmp_data_subset.csv")


##############
########### heatmaps2
#############################

## count number of sig compounds
fdr.p <- p.adjust(mSet$analSet$aov$p.value, "fdr")
fdr.p <- as.data.frame(fdr.p)
numb.fdr.p<-NROW(dplyr::filter(fdr.p,fdr.p < 0.1))
#numb.fdr.p <- 75 ## or make static number
print ("Number of signifcant compounds FDR 0.1 :")
print (numb.fdr.p)


if(numb.fdr.p > 0) {
  var.nms <- names(sort(mSet$analSet$aov$p.value))[1:numb.fdr.p]  ## reports the x signficant compounds
  var.inx <- match(var.nms, colnames(mSet$dataSet$norm))
  my.data <- mSet$dataSet$norm
  hc.dat <- as.matrix(my.data[, var.inx])

  colors <- rev(colorRampPalette(RColorBrewer::brewer.pal(10, "RdBu"))(256))

  clusters<-pheatmap::pheatmap(t(hc.dat),
                               fontsize = 5,clustering_distance_rows = "euclidean",
                               clustering_distance_cols = "euclidean", clustering_method = "ward.D",
                               border_color = "grey", cluster_rows = T, cluster_cols = T, scale = "row"
                               cellheight = 4.402, cellwidth = 11.709, annotation_colors = TRUE, color =
                               filename = paste("analysisANOVA/heatmap/heatmapSigONLY_",dataname[1],data
```

```
                             main =  paste(dataname[1],dataname[length(dataname)],sep='\n'))
  ###################################



  ###############


  colortable <- read.table("H:/DusLab/data/Metabolimics/Data/Data3/colorCompoundNames/colorClassKey.txt
  NameIDClasstable <- read.table("H:/DusLab/data/Metabolimics/Data/Data3/colorCompoundNames/compoundsNa

  namesforheatmap <- clusters$tree_row$labels[clusters$tree_row$order]  ## puts IDs in order
  namesforheatmap <-  as.data.frame(namesforheatmap)  ## convert to data frame
  names (namesforheatmap) <- "COMP_ID"  ## add a header name

  namesforheatmap <- dplyr::inner_join(namesforheatmap,NameIDClasstable, by="COMP_ID")  ## only the comp
  namesforheatmap <- dplyr::inner_join(namesforheatmap,colortable, by = "SUPER_PATHWAY")  ## add color

  x <- namesforheatmap$BIOCHEMICAL ## take only BIOCHEMICAL (biochemical names)
  y <- c(1:numb.fdr.p)  ## dummy data

  a <- namesforheatmap$color

  plot_data <- data.frame(x, y)

  #plot_data$y <- as.numeric(as.character(plot_data$y))
  #plot_data <- t(plot_data)
  plot_data <-plot_data[dim(plot_data)[1]:1,] ## flip order
  a <-rev(a) ## flip order
  p <- ggplot(plot_data, aes(x = y, y = x)) +
    #geom_bar(stat = "identity") +
    geom_point() +
    scale_y_discrete(limits=plot_data$x) +  ## this prevents re-sorting
    theme(axis.text.y = element_text(angle = 0, hjust = 1, color = a, size = 4)) +
    ggtitle(paste(dataname[1],dataname[length(dataname)],sep='\n')) +
    xlab("toy data") +
    ylab("Names")

  ggsave(paste("analysisANOVA/heatmap/heatmapNamesColored_",dataname[1],dataname[length(dataname)],".pd

}
}
```

```
## [1] "R objects intialized ..."

## [1] "A total of 296 significant features were found."

## [1] "Number of signifcant compounds FDR 0.1 :"
## [1] 194

## [1] "R objects intialized ..."

## [1] "A total of 276 significant features were found."

## [1] "Number of signifcant compounds FDR 0.1 :"
## [1] 161
```

```r
orderedbycluster <-  rownames(t(hc.dat)[clusters$tree_row[["order"]],])
  ## add data

  orderedbycluster <- as.data.frame.integer(orderedbycluster)
  colnames(orderedbycluster) <- "ID"
  head(orderedbycluster)
```

```
##      ID
## 1  1898
## 2 34398
## 3  1437
## 4 37063
## 5  1124
## 6 34409
```

```r
  hc.dat <- (t(hc.dat))
  hc.dat <- as.data.frame(hc.dat)
  hc.dat <- rownames_to_column(hc.dat, var="ID")
  head(hc.dat)
```

```
##      ID a2D_HSD_starved_heads_46 a2D_HSD_starved_heads_47
## 1   443               -0.1728428              -0.14359894
## 2  2730               -0.3020037              -0.27682186
## 3   528               -0.1684700              -0.34638619
## 4  1643               -0.3030727              -0.20315288
## 5  1303               -0.3117996              -0.20414891
## 6 42002                0.3162049               0.05929058
##   a2D_HSD_starved_heads_48 a2D_HSD_starved_heads_49
## 1              -0.09895506             -0.176892735
## 2              -0.31118642             -0.239647306
## 3              -0.39026832              0.001582905
## 4              -0.26073366             -0.265421748
## 5              -0.29571733             -0.290409850
## 6               0.38839610             -0.059104350
##   a2D_HSD_starved_heads_50 a5D_HSD_starved_heads_36
## 1              -0.16546694              -0.17410934
## 2              -0.04816475              -0.24312742
## 3              -0.11481845               0.07689252
## 4              -0.11200641              -0.26435864
## 5              -0.07295410              -0.26821532
## 6              -0.17509488               0.67536415
##   a5D_HSD_starved_heads_37 a5D_HSD_starved_heads_38
## 1              -0.16611664               -0.1836369
## 2              -0.27156232               -0.2968839
## 3              -0.05426125               -0.1226896
## 4              -0.20310416               -0.2831909
## 5              -0.21220910               -0.2870056
## 6               0.50615046                0.2086612
##   a5D_HSD_starved_heads_39 a7D_HSD_starved_heads_16
## 1               -0.1693590             -0.005478764
## 2               -0.2373643              0.159419447
## 3               -0.1304409              0.609731683
## 4               -0.2401268              0.102311555
## 5               -0.2527088              0.113206266
```

```
## 6                0.4688693             -0.324635849
##   a7D_HSD_starved_heads_17 a7D_HSD_starved_heads_18
## 1              -0.13255520               -0.1337317
## 2               0.05691252                0.2871835
## 3               0.52939804                0.4608432
## 4               0.31811407                0.5513006
## 5               0.27382943                0.5573779
## 6              -0.32463585               -0.2072926
##   a7D_HSD_starved_heads_19 a7D_HSD_starved_heads_20 a7D_ND_starved_heads_1
## 1              -0.12859849              -0.14588918             0.61780671
## 2               0.08541038              -0.03533759             0.38701026
## 3               0.24304819               0.50538878            -0.37663334
## 4               0.69692730               0.54161350            -0.07937683
## 5               0.63841366               0.68820041            -0.07142693
## 6              -0.32463585              -0.24567958            -0.32463585
##   a7D_ND_starved_heads_2 a7D_ND_starved_heads_3
## 1            0.563061952             0.81636305
## 2            0.688813583             0.59734977
## 3           -0.358466412            -0.36445095
## 4           -0.007503021             0.01178065
## 5           -0.026995208             0.02256298
## 6           -0.324635849            -0.31258612
```

```
hc.dat2 <- dplyr::inner_join(orderedbycluster,hc.dat,by="ID")
head(hc.dat2)
```

```
##      ID a2D_HSD_starved_heads_46 a2D_HSD_starved_heads_47
## 1  1898             -0.277904028              -0.25393925
## 2 34398             -0.265825463              -0.26582546
## 3  1437             -0.417687162              -0.29171593
## 4 37063             -0.219964596              -0.27359105
## 5  1124              0.007957584              -0.07249855
## 6 34409             -0.147918636              -0.21484264
##   a2D_HSD_starved_heads_48 a2D_HSD_starved_heads_49
## 1              -0.33434691              -0.02327213
## 2              -0.26582546               0.03888608
## 3              -0.19799695              -0.14285043
## 4              -0.25875410               0.11947575
## 5              -0.15240503               0.06995387
## 6               0.03300359               0.20893205
##   a2D_HSD_starved_heads_50 a5D_HSD_starved_heads_36
## 1               0.03929905              0.074909353
## 2               0.24843432              0.136287794
## 3               0.25269840             -0.118254405
## 4               0.39196724             -0.170546939
## 5               0.04430222              0.093854743
## 6               0.07352106              0.005360887
##   a5D_HSD_starved_heads_37 a5D_HSD_starved_heads_38
## 1              -0.19020717               -0.2898354
## 2              -0.04150528               -0.2658255
## 3              -0.24754034               -0.4128003
## 4              -0.21809241               -0.1688492
## 5              -0.11710309               -0.3003844
## 6              -0.13375522               -0.2696319
##   a5D_HSD_starved_heads_39 a7D_HSD_starved_heads_16
```

```
## 1             -0.009689419              0.214495861
## 2             -0.265825463              0.002983666
## 3             -0.098797971              0.346529306
## 4             -0.167747880              0.155456606
## 5             -0.112590229             -0.163059713
## 6             -0.175947434             -0.114531990
##   a7D_HSD_starved_heads_17 a7D_HSD_starved_heads_18
## 1               0.27682113                0.2874203
## 2              -0.05015858                0.5628068
## 3               0.03305987                0.5823128
## 4               0.08261241                0.7264090
## 5              -0.12559616                0.6505748
## 6              -0.06722408                0.6184366
##   a7D_HSD_starved_heads_19 a7D_HSD_starved_heads_20 a7D_ND_starved_heads_1
## 1                0.6656531                0.6292415             -0.2932715
## 2                0.4946944                0.7341745             -0.2658255
## 3                0.4801918                0.3943653             -0.1350361
## 4                0.4426294                0.1610358             -0.2222147
## 5                0.6196118                0.4254092             -0.3494252
## 6                0.5572126                0.3614529             -0.1783165
##   a7D_ND_starved_heads_2 a7D_ND_starved_heads_3
## 1            -0.23724376            -0.27813068
## 2            -0.26582546            -0.26582546
## 3             0.05493536            -0.08141329
## 4            -0.21160974            -0.16821553
## 5            -0.34722437            -0.17137735
## 6            -0.17418801            -0.38156335
```

```
newDF <- hc.dat2 %>%
  mutate(mean_0ND = rowMeans(select(.,a7D_ND_starved_heads_1:a7D_ND_starved_heads_3))) %>%
  mutate(mean_2D = rowMeans(select(.,a2D_HSD_starved_heads_46:a2D_HSD_starved_heads_50))) %>%
  mutate(mean_5D = rowMeans(select(.,a5D_HSD_starved_heads_36:a5D_HSD_starved_heads_39))) %>%
  mutate(mean_7D = rowMeans(select(.,a7D_HSD_starved_heads_16:a7D_HSD_starved_heads_20)))


head(newDF)
```

```
##      ID a2D_HSD_starved_heads_46 a2D_HSD_starved_heads_47
## 1  1898             -0.277904028              -0.25393925
## 2 34398             -0.265825463              -0.26582546
## 3  1437             -0.417687162              -0.29171593
## 4 37063             -0.219964596              -0.27359105
## 5  1124              0.007957584              -0.07249855
## 6 34409             -0.147918636              -0.21484264
##   a2D_HSD_starved_heads_48 a2D_HSD_starved_heads_49
## 1              -0.33434691              -0.02327213
## 2              -0.26582546               0.03888608
## 3              -0.19799695              -0.14285043
## 4              -0.25875410               0.11947575
## 5              -0.15240503               0.06995387
## 6               0.03300359               0.20893205
##   a2D_HSD_starved_heads_50 a5D_HSD_starved_heads_36
## 1               0.03929905              0.074909353
## 2               0.24843432              0.136287794
## 3               0.25269840             -0.118254405
```

```
## 4              0.39196724             -0.170546939
## 5              0.04430222              0.093854743
## 6              0.07352106              0.005360887
##   a5D_HSD_starved_heads_37 a5D_HSD_starved_heads_38
## 1              -0.19020717               -0.2898354
## 2              -0.04150528               -0.2658255
## 3              -0.24754034               -0.4128003
## 4              -0.21809241               -0.1688492
## 5              -0.11710309               -0.3003844
## 6              -0.13375522               -0.2696319
##   a5D_HSD_starved_heads_39 a7D_HSD_starved_heads_16
## 1             -0.009689419              0.214495861
## 2             -0.265825463              0.002983666
## 3             -0.098797971              0.346529306
## 4             -0.167747880              0.155456606
## 5             -0.112590229             -0.163059713
## 6             -0.175947434             -0.114531990
##   a7D_HSD_starved_heads_17 a7D_HSD_starved_heads_18
## 1               0.27682113                0.2874203
## 2              -0.05015858                0.5628068
## 3               0.03305987                0.5823128
## 4               0.08261241                0.7264090
## 5              -0.12559616                0.6505748
## 6              -0.06722408                0.6184366
##   a7D_HSD_starved_heads_19 a7D_HSD_starved_heads_20 a7D_ND_starved_heads_1
## 1                0.6656531                0.6292415             -0.2932715
## 2                0.4946944                0.7341745             -0.2658255
## 3                0.4801918                0.3943653             -0.1350361
## 4                0.4426294                0.1610358             -0.2222147
## 5                0.6196118                0.4254092             -0.3494252
## 6                0.5572126                0.3614529             -0.1783165
##   a7D_ND_starved_heads_2 a7D_ND_starved_heads_3    mean_0ND      mean_2D
## 1            -0.23724376            -0.27813068 -0.26954863 -0.170032652
## 2            -0.26582546            -0.26582546 -0.26582546 -0.102031198
## 3             0.05493536            -0.08141329 -0.05383799 -0.159510415
## 4            -0.21160974            -0.16821553 -0.20067998 -0.048173351
## 5            -0.34722437            -0.17137735 -0.28934231 -0.020537982
## 6            -0.17418801            -0.38156335 -0.24468928 -0.009460915
##      mean_5D   mean_7D
## 1 -0.1037057 0.4147264
## 2 -0.1092171 0.3489002
## 3 -0.2193483 0.3672918
## 4 -0.1813091 0.3136286
## 5 -0.1090557 0.2813880
## 6 -0.1434934 0.2710692
```

```
newDF <- newDF %>%
  select(ID,mean_0ND,mean_2D,mean_5D,mean_7D)  ## make a new dataframe that only has ave numbers

head(newDF)
```

```
##      ID    mean_0ND      mean_2D    mean_5D   mean_7D
## 1  1898 -0.26954863 -0.170032652 -0.1037057 0.4147264
## 2 34398 -0.26582546 -0.102031198 -0.1092171 0.3489002
## 3  1437 -0.05383799 -0.159510415 -0.2193483 0.3672918
```

```
## 4 37063 -0.20067998 -0.048173351 -0.1813091 0.3136286
## 5  1124 -0.28934231 -0.020537982 -0.1090557 0.2813880
## 6 34409 -0.24468928 -0.009460915 -0.1434934 0.2710692
```

```r
rownames(newDF) <- newDF[,1] ## make row 1 names
newDF[,1] <- NULL  ## remove IDs
head(newDF)
```

```
##          mean_0ND     mean_2D    mean_5D   mean_7D
## 1898  -0.26954863 -0.170032652 -0.1037057 0.4147264
## 34398 -0.26582546 -0.102031198 -0.1092171 0.3489002
## 1437  -0.05383799 -0.159510415 -0.2193483 0.3672918
## 37063 -0.20067998 -0.048173351 -0.1813091 0.3136286
## 1124  -0.28934231 -0.020537982 -0.1090557 0.2813880
## 34409 -0.24468928 -0.009460915 -0.1434934 0.2710692
```

```r
include_list <- c("18374", "1604", "37465")
plottingdata <- data.frame(t(newDF[include_list, ]))

plottingdata <- rownames_to_column(plottingdata, var="State")

## add color names




#  x <- plottingdata$State ## Diets
#  y <-  plottingdata$`X18374` ## Compound ID
#  plot_data <- data.frame(x, y)

#ggplot(data=plottingdata,aes(y=X18374,x=State,group=1)) +
#  geom_line()+
#  geom_point()



library(reshape2)
long <- melt(plottingdata)
head(long)
```

```
##      State variable       value
## 1 mean_0ND   X18374 -0.07781772
## 2  mean_2D   X18374  0.21470663
## 3  mean_5D   X18374  0.17071984
## 4  mean_7D   X18374 -0.30459188
## 5 mean_0ND    X1604 -0.25155065
## 6  mean_2D    X1604  0.15870252
```

```r
ggplot(long, aes(x=State, y=value, group=variable)) +
    geom_line()

#Add colors based on compound class
```

## Supplementary Note 3

R code used for statistical analysis of RNA-sequencing data.

# RNA-sequencing analysis by DESeq2

Load libraries make directories

```r
library("DESeq2")
```

```
## Loading required package: S4Vectors

## Loading required package: stats4

## Loading required package: BiocGenerics

## Loading required package: parallel

##
## Attaching package: 'BiocGenerics'

## The following objects are masked from 'package:parallel':
##
##     clusterApply, clusterApplyLB, clusterCall, clusterEvalQ,
##     clusterExport, clusterMap, parApply, parCapply, parLapply,
##     parLapplyLB, parRapply, parSapply, parSapplyLB

## The following objects are masked from 'package:stats':
##
##     IQR, mad, sd, var, xtabs

## The following objects are masked from 'package:base':
##
##     anyDuplicated, append, as.data.frame, basename, cbind,
##     colMeans, colnames, colSums, dirname, do.call, duplicated,
##     eval, evalq, Filter, Find, get, grep, grepl, intersect,
##     is.unsorted, lapply, lengths, Map, mapply, match, mget, order,
##     paste, pmax, pmax.int, pmin, pmin.int, Position, rank, rbind,
##     Reduce, rowMeans, rownames, rowSums, sapply, setdiff, sort,
##     table, tapply, union, unique, unsplit, which, which.max,
##     which.min

##
## Attaching package: 'S4Vectors'

## The following object is masked from 'package:base':
##
##     expand.grid

## Loading required package: IRanges

##
## Attaching package: 'IRanges'

## The following object is masked from 'package:grDevices':
##
##     windows

## Loading required package: GenomicRanges

## Loading required package: GenomeInfoDb
```

```
## Loading required package: SummarizedExperiment

## Loading required package: Biobase

## Welcome to Bioconductor
##
##     Vignettes contain introductory material; view with
##     'browseVignettes()'. To cite Bioconductor, see
##     'citation("Biobase")', and for packages 'citation("pkgname")'.

## Loading required package: DelayedArray

## Loading required package: matrixStats

##
## Attaching package: 'matrixStats'

## The following objects are masked from 'package:Biobase':
##
##     anyMissing, rowMedians

## Loading required package: BiocParallel

##
## Attaching package: 'DelayedArray'

## The following objects are masked from 'package:matrixStats':
##
##     colMaxs, colMins, colRanges, rowMaxs, rowMins, rowRanges

## The following objects are masked from 'package:base':
##
##     aperm, apply
```

```r
library("ggplot2")
library("tibble")
library("dplyr")
```

```
##
## Attaching package: 'dplyr'

## The following object is masked from 'package:matrixStats':
##
##     count

## The following object is masked from 'package:Biobase':
##
##     combine

## The following objects are masked from 'package:GenomicRanges':
##
##     intersect, setdiff, union

## The following object is masked from 'package:GenomeInfoDb':
##
##     intersect

## The following objects are masked from 'package:IRanges':
##
##     collapse, desc, intersect, setdiff, slice, union

## The following objects are masked from 'package:S4Vectors':
##
```

```
##     first, intersect, rename, setdiff, setequal, union

## The following objects are masked from 'package:BiocGenerics':
##
##     combine, intersect, setdiff, union

## The following objects are masked from 'package:stats':
##
##     filter, lag

## The following objects are masked from 'package:base':
##
##     intersect, setdiff, setequal, union
```

```
#library("pheatmap")
#library("RColorBrewer")
library(EnhancedVolcano)
```

```
## Loading required package: ggrepel
```

```
dir.create("analysis")
```

```
## Warning in dir.create("analysis"): 'analysis' already exists
```

```
dir.create("analysis/plots")
```

```
## Warning in dir.create("analysis/plots"): 'analysis\plots' already exists
```

Load data

```
FILES_CD_brains_refedVsfasted <- c(
"91819_AGTGAG_S18_R1_ReadsPerGene.out.tab", ## CD refed
"91826_AGCATG_S25_R1_ReadsPerGene.out.tab", ## CD refed
"91827_GAGTCA_S26_R1_ReadsPerGene.out.tab", ## CD refed
"91822_GTGCTT_S21_R1_ReadsPerGene.out.tab", ## CD fasted
"91823_AAGCCT_S22_R1_ReadsPerGene.out.tab", ## CD fasted
"91830_CACAGT_S29_R1_ReadsPerGene.out.tab"  ## CD fasted
)
FILES_CD_brains_satedVsfassted <- c(
  "91820_GCACTA_S19_R1_ReadsPerGene.out.tab", ## CD sated
  "91821_ACCTCA_S20_R1_ReadsPerGene.out.tab", ## CD sated
  "91828_CGTAGA_S27_R1_ReadsPerGene.out.tab", ## CD sated
  "91822_GTGCTT_S21_R1_ReadsPerGene.out.tab", ## CD fasted
  "91823_AAGCCT_S22_R1_ReadsPerGene.out.tab", ## CD fasted
  "91830_CACAGT_S29_R1_ReadsPerGene.out.tab"  ## CD fasted
)
FILES_CD_brains_refedVssated <- c(
"91819_AGTGAG_S18_R1_ReadsPerGene.out.tab", ## CD refed
"91826_AGCATG_S25_R1_ReadsPerGene.out.tab", ## CD refed
"91827_GAGTCA_S26_R1_ReadsPerGene.out.tab", ## CD refed
"91820_GCACTA_S19_R1_ReadsPerGene.out.tab", ## CD sated
"91821_ACCTCA_S20_R1_ReadsPerGene.out.tab", ## CD sated
"91828_CGTAGA_S27_R1_ReadsPerGene.out.tab" ## CD sated
)
```

initalize data.frames

```
CD_brains_refedVsfasted <- read.table(FILES_CD_brains_refedVsfasted[1])
CD_brains_satedVsfassted <- read.table(FILES_CD_brains_satedVsfassted[1])
```

```
CD_brains_refedVssated<- read.table(FILES_CD_brains_refedVssated[1])
```

keep only IDs

```
CD_brains_refedVsfasted <- dplyr::select(CD_brains_refedVsfasted,"V1")
CD_brains_satedVsfassted <- dplyr::select(CD_brains_satedVsfassted,"V1")
CD_brains_refedVssated <- dplyr::select(CD_brains_refedVssated,"V1")
```

rename column name

```
colnames(CD_brains_refedVsfasted) <- c("ID")
colnames(CD_brains_satedVsfassted) <- c("ID")
colnames(CD_brains_refedVssated) <- c("ID")
```

Read data from files in loop (CD__brains__refedVsfasted)

```
for(file_name in FILES_CD_brains_refedVsfasted) {
  tmp <- read.table(file_name)
  colnames(tmp) <- c("ID","one",file_name,"three")
  tmp <- dplyr::select(tmp,"ID",file_name)
  CD_brains_refedVsfasted <- dplyr::full_join(CD_brains_refedVsfasted,tmp,by="ID")
}
```

remove following rows

```
CD_brains_refedVsfasted <-  CD_brains_refedVsfasted %>%
  filter(ID!="N_unmapped") %>%
  filter(ID!="N_multimapping") %>%
  filter(ID!="N_noFeature") %>%
  filter(ID!="N_ambiguous")
```

Read data from files in loop (CD__brains__satedVsfassted)

```
for(file_name in FILES_CD_brains_satedVsfassted) {
  tmp <- read.table(file_name)
  colnames(tmp) <- c("ID","one",file_name,"three")
  tmp <- dplyr::select(tmp,"ID",file_name)
  CD_brains_satedVsfassted <- dplyr::full_join(CD_brains_satedVsfassted,tmp,by="ID")
}
```

remove following rows

```
CD_brains_satedVsfassted <-  CD_brains_satedVsfassted %>%
  filter(ID!="N_unmapped") %>%
  filter(ID!="N_multimapping") %>%
  filter(ID!="N_noFeature") %>%
  filter(ID!="N_ambiguous")
```

Read data from files in loop (CD__brains__refedVssated)

```
for(file_name in FILES_CD_brains_refedVssated) {
  tmp <- read.table(file_name)
  colnames(tmp) <- c("ID","one",file_name,"three")
  tmp <- dplyr::select(tmp,"ID",file_name)
  CD_brains_refedVssated <- dplyr::full_join(CD_brains_refedVssated,tmp,by="ID")
}
```

remove following rows

```
CD_brains_refedVssated <-  CD_brains_refedVssated %>%
  filter(ID!="N_unmapped") %>%
  filter(ID!="N_multimapping") %>%
  filter(ID!="N_noFeature") %>%
  filter(ID!="N_ambiguous")
```

Make ID row names

```
rownames(CD_brains_refedVsfasted) <- CD_brains_refedVsfasted[,1]
CD_brains_refedVsfasted[,1] <- NULL  ## remove IDs
rownames(CD_brains_satedVsfassted) <- CD_brains_satedVsfassted[,1]
CD_brains_satedVsfassted[,1] <- NULL  ## remove IDs
rownames(CD_brains_refedVssated) <- CD_brains_refedVssated[,1]
CD_brains_refedVssated[,1] <- NULL  ## remove IDs
```

```
listofcountdatas <- list(
  CD_brains_refedVsfasted,
  CD_brains_satedVsfassted,
  CD_brains_refedVssated
  )
```

Make column data file

```
condition <- c(
  "CD_refed",
  "CD_refed",
  "CD_refed",
  "CD_fasted",
  "CD_fasted",
  "CD_fasted"
)
CD_brains_refedVsfasted_colData <- as.data.frame(cbind(FILES_CD_brains_refedVsfasted,condition))

condition <- c(
  "CD_sated",
  "CD_sated",
  "CD_sated",
  "CD_fasted",
  "CD_fasted",
  "CD_fasted"
)
CD_brains_satedVsfassted_colData <- as.data.frame(cbind(FILES_CD_brains_satedVsfassted,condition))

condition <- c(
  "CD_refed",
  "CD_refed",
  "CD_refed",
  "CD_sated",
  "CD_sated",
  "CD_sated"
)
CD_brains_refedVssated_colData <- as.data.frame(cbind(FILES_CD_brains_satedVsfassted,condition))

#rownames(CD_brains_refedVsfasted_colData) <- CD_brains_refedVsfasted_colData[,1]
#CD_brains_refedVsfasted_colData[,1] <- NULL  ## remove IDs
```

```
#rownames(CD_brains_satedVsfassted_colData) <- CD_brains_satedVsfassted_colData[,1]
#CD_brains_satedVsfassted_colData[,1] <- NULL  ## remove IDs
```

```
listofcoldatas <- list(
  CD_brains_refedVsfasted_colData,
  CD_brains_satedVsfassted_colData,
  CD_brains_refedVssated_colData
)
```

### DeSeq2

```
i<-0
for(dataname in listofcountdatas) {
  i<-i+1
  countData <- dataname
  colData <- as.data.frame(listofcoldatas[i])

  head(countData)
  colData
  rm(dds)
  dds <- DESeqDataSetFromMatrix(countData = countData,
                                colData = colData,
                                design = ~ condition)

  #filter rows with fewer than 10 counts per gene
  dim(dds)
  keep <- rowSums(counts(dds)) >= 10
  dds <- dds[keep,]
  dim (dds)

  #differental aboundance
  dds <- DESeq(dds)
  res <- results(dds)
  #reorder based on padj
  res <- res[order(res$padj),]
  rld <- rlog(dds, blind=FALSE)
  #save data
  write.csv(as.data.frame(counts(dds,normalized=TRUE)), file= paste("analysis/Norm_counts_",colData$cond[illegible]
  #write.csv(as.data.frame(res), file=paste("analysis/Results_",i,".csv",sep=''))

  ## PCA plot with data lables
  #pdf(paste("PCA_",i,".pdf",sep=''))
  #pcaData <- plotPCA(rld,intgroup="condition", ntop=5000, returnData=TRUE)
  #ggplot(pcaData, aes(PC1, PC2, label = rownames(pcaData), color=condition)) +
  #geom_point(size=3) +
  #geom_text() +
  #labs(title=dataname)

  #plotPCA(rld,intgroup="condition",ntop=500) ## simple PCA plot w/o data labeles
  #print(p)
  #ggsave(paste("PCA_",i,".pdf",sep=''), plot = p, width= 10, height = 10)

  ## MA plot
```

```
  pdf(paste("analysis/plots/MAplot_",colData$condition[1],"Vs",colData$condition[4],i,".pdf",sep=''))
  plotMA(res, ylim=c(-5,5))
  dev.off()

  ## Volcano plot
  IDtoNames <- read.table("IDtoNames.txt",header=T)
  res2 <- as.data.frame(res)
  res2 <- rownames_to_column(res2,var = "ID")
  res2 <- dplyr::full_join(res2,IDtoNames,by="ID")
  res3 <- na.omit(res2)
  write.csv(as.data.frame(res3), file=paste("analysis/Results_",colData$condition[1],"Vs",colData$condi


  p <- EnhancedVolcano(res2,
              lab=res2$Name,x="log2FoldChange",
              y="padj",
              title = paste(colData$condition[1]," Vs ",colData$condition[4],sep=''),
              pCutoff = 0.05,
              FCcutoff = 1.5,
              legendPosition = 'bottom',
              colAlpha = 1,
              DrawConnectors = TRUE,
              widthConnectors =  0.2,
              colConnectors = 'grey30'
              )
  ggsave(paste("analysis/plots/volcano_",colData$condition[1],"Vs",colData$condition[4],i,".pdf",sep=''
}
```

```
## Warning in rm(dds): object 'dds' not found

## estimating size factors

## estimating dispersions

## gene-wise dispersion estimates

## mean-dispersion relationship

## final dispersion estimates

## fitting model and testing

## Warning: Column `ID` joining character vector and factor, coercing into
## character vector

## Warning: Removed 1777 rows containing missing values (geom_point).

## estimating size factors

## estimating dispersions

## gene-wise dispersion estimates

## mean-dispersion relationship

## -- note: fitType='parametric', but the dispersion trend was not well captured by the
##    function: y = a/x + b, and a local regression fit was automatically substituted.
##    specify fitType='local' or 'mean' to avoid this message next time.

## final dispersion estimates
```

```
## fitting model and testing
## Warning: Column `ID` joining character vector and factor, coercing into
## character vector
## Warning: Removed 3322 rows containing missing values (geom_point).
## estimating size factors
## estimating dispersions
## gene-wise dispersion estimates
## mean-dispersion relationship
## final dispersion estimates
## fitting model and testing
## Warning: Column `ID` joining character vector and factor, coercing into
## character vector
## Warning: Removed 226 rows containing missing values (geom_point).
## Warning: Removed 1 rows containing missing values (geom_text_repel).
```

References

1 Dobson, A. J. *et al.* Nutritional Programming of Lifespan by FOXO Inhibition on Sugar-Rich Diets. *Cell Rep* **18**, 299-306, doi:10.1016/j.celrep.2016.12.029 (2017).
