# Supplementary material for: Rapid metabolic shifts occur during the transition between hunger and satiety in Drosophila melanogaster
Source: Nat Commun. 2019 Sep 6;10:4052. doi: 10.1038/s41467-019-11933-z (PMC6731244; doi:10.1038/s41467-019-11933-z)
Supplement: Supplementary file 3 — Description of Additional Supplementary Files [file 41467_2019_11933_MOESM3_ESM.docx]

**Description of Supplementary Files**

**File Name: Supplementary Data 1**

**Description:** Metabolomics data used for compound abundance analyses and comparisons. Green shows metabolites with FDR < 0.1, and blue shows metabolites with FDR < 0.05.

**File Name: Supplementary Data 2**

**Description:** RNA sequencing data used for RNA abundance analyses and comparisons.

**File Name: Supplementary Data 3**

**Description:** FLIC summary data used for feeding behavior analyses and comparisons.

**File Name: Supplementary Data 4**

**Description:** Raw FLIC data used for feeding behavior analyses and comparisons.
