# Supplementary material for: Rapid metabolic shifts occur during the transition between hunger and satiety in Drosophila melanogaster
Source: Nat Commun. 2019 Sep 6;10:4052. doi: 10.1038/s41467-019-11933-z (PMC6731244; doi:10.1038/s41467-019-11933-z)
Supplement: Supplementary file 8 — Reporting Summary [file 41467_2019_11933_MOESM8_ESM.pdf]

nature research

Corresponding author(s): Monica Dus

Reporting Summary

Nature Research wishes to improve the reproducibility of the work that we publish. This form provides structure for consistency and transparency in reporting. For further information on Nature Research policies, see Authors & Referees and the Editorial Policy Checklist.

Statistical parameters

When statistical analyses are reported, confirm that the following items are present in the relevant location (e.g. figure legend, table legend, main text, or Methods section).

n/a	Confirmed	
☐	☒	The exact sample size (*n*) for each experimental group/condition, given as a discrete number and unit of measurement
☐	☒	An indication of whether measurements were taken from distinct samples or whether the same sample was measured repeatedly
☐	☒	The statistical test(s) used AND whether they are one- or two-sided<br>*Only common tests should be described solely by name; describe more complex techniques in the Methods section.*
☒	☐	A description of all covariates tested
☐	☒	A description of any assumptions or corrections, such as tests of normality and adjustment for multiple comparisons
☐	☒	A full description of the statistics including central tendency (e.g. means) or other basic estimates (e.g. regression coefficient) AND variation (e.g. standard deviation) or associated estimates of uncertainty (e.g. confidence intervals)
☐	☒	For null hypothesis testing, the test statistic (e.g. *F*, *t*, *r*) with confidence intervals, effect sizes, degrees of freedom and *P* value noted<br>*Give P values as exact values whenever suitable.*
☐	☒	For Bayesian analysis, information on the choice of priors and Markov chain Monte Carlo settings
☒	☐	For hierarchical and complex designs, identification of the appropriate level for tests and full reporting of outcomes
☒	☐	Estimates of effect sizes (e.g. Cohen's *d*, Pearson's *r*), indicating how they were calculated
☐	☒	Clearly defined error bars<br>*State explicitly what error bars represent (e.g. SD, SE, CI)*

Our web collection on statistics for biologists may be useful.

Software and code

Policy information about availability of computer code

Data collection	Mass spectrometry raw data was extracted, peak-identified, and QC processed using Metabolon (Durham, NC) hardware and software.<br>RNA-sequencing data was collected using Illumina (San Diego, CA) NextSeq-500 software.
Data analysis	All statistical analysis of metabolomics data was conducted in the R version of MetaboAnalyst (MetaboAnalystR_1.0.2, Windows 10 x64, R version 3.5.2) https://github.com/xia-lab/MetaboAnalystR.<br>The Drosophila melanogaster (BDGP6) genomic sequence was downloaded from Ensembl (http://useast.ensembl.org/Drosophila_melanogaster/Info/Index).<br>Sequencing reads were aligned with STAR (2.5.2a) (https://github.com/alexdobin/STAR) using –quantMode GeneCounts and differential abundance calls were made by DESeq2 (1.18.1, R version 3.4.3) (http://bioconductor.org/packages/release/bioc/html/DESeq2.html).<br>DAVID (6.8) was used for Gene Ontology enrichment analysis (https://david.ncifcrf.gov/).<br>Flyscape (0.6.0, Cytoscape version 3.4.0) was used to generate network representations (https://apps.cytoscape.org/apps/flyscape).<br>Behavior data were analyzed using GraphPad Prism 8.0.0.<br>R code used for analysis (FLIC, metabolomics, and RNA sequencing) is included as R markdown files (Supplementary Note 1-3).

For manuscripts utilizing custom algorithms or software that are central to the research but not yet described in published literature, software must be made available to editors/reviewers upon request. We strongly encourage code deposition in a community repository (e.g. GitHub). See the Nature Research guidelines for submitting code & software for further information.

Data

Policy information about availability of data

All manuscripts must include a data availability statement. This statement should provide the following information, where applicable:

- Accession codes, unique identifiers, or web links for publicly available datasets
- A list of figures that have associated raw data
- A description of any restrictions on data availability

RNA sequencing reads will be deposited in Gene Expression Omnibus before publication and the accession number listed in the data availability statement of the manuscript. Raw and processed metabolomics data are in Supplementary Table 1. Metabolomics and RNA-sequencing data will be available for download without restriction within limits of the copyright. Flyscape is available from the Cytoscape App Store https://apps.cytoscape.org/apps/flyscape.

Field-specific reporting

Please select the best fit for your research. If you are not sure, read the appropriate sections before making your selection.

☒ Life sciences ☐ Behavioural & social sciences ☐ Ecological, evolutionary & environmental sciences

For a reference copy of the document with all sections, see nature.com/authors/policies/ReportingSummary-flat.pdf

Life sciences study design

All studies must disclose on these points even when the disclosure is negative.

Sample size	We choose five biological replicates (groups of 100 heads or 50 bodies) to collect for mass spectrometry because 3-5 is the convention in the field.<br>We used 10 brains per RNA sequencing replicate. Based on test experiments, 10 brains was the lowest number that consistently yielded enough total RNA for subsequent sample preparation. Four biological replicates were chosen because it is the convention in the field.
Data exclusions	For mass spectrometry, all experimental conditions included 5 biological replicates. One sample (CD fasted heads) was lost during preparation. Four (control diet fasted bodies, control diet fasted heads, sugar diet 5 days refed heads, sugar diet 5 days fasted heads) samples were determined outliers by principle component analysis and removed from analysis.<br>For RNA-sequencing, all experimental conditions included 4 biological replicates. One (CD refed) sample failed during sample preparation and was not sequenced. One (CD sated) sample failed to sequence. One (CD starved) sample was determined an outlier by principle component analysis and removed from further analysis.
Replication	All experiments were done in triplicate or more and represented in the manuscript.
Randomization	Flies with the same genotype (w1118CS) were allocated into experimental groups at random. Mass spectrometry samples were randomly loaded onto the instruments and RNA-seq samples were sequenced together in a pool.
Blinding	Investigators were not blinded to group allocation during data collection and analysis. Blinding is not relevant to this study as the molecular methods used in this study are not typically sensitive to collection bias.

Reporting for specific materials, systems and methods

Materials & experimental systems

n/a	Involved in the study	
☒	☐	Unique biological materials
☒	☐	Antibodies
☒	☐	Eukaryotic cell lines
☒	☐	Palaeontology
☐	☒	Animals and other organisms
☒	☐	Human research participants

Methods

n/a	Involved in the study	
☒	☐	ChIP-seq
☒	☐	Flow cytometry
☒	☐	MRI-based neuroimaging

Animals and other organisms

Policy information about studies involving animals; ARRIVE guidelines recommended for reporting animal research

Laboratory animals	This study used male Drosophila melanogaster line w1118CS obtained from Ann Simon, University of Western Ontario, Canada.
Wild animals	This study did not involve wild animals.
Field-collected samples	This study did not involve samples collected from the field.
